# The importance of anchoring ligands of binuclear sensitizers on electron transfer processes and photovoltaic action in dye-sensitized solar cells

**DOI:** 10.1038/s41598-023-44015-8

**Published:** 2023-10-05

**Authors:** Aleksandra Bartkowiak, Oleksandr Korolevych, Błażej Gierczyk, Daniel Pelczarski, Alberto Bossi, Maciej Klein, Łukasz Popenda, Waldemar Stampor, Malgorzata Makowska-Janusik, Maciej Zalas

**Affiliations:** 1 Faculty of Chemistry, Adam Mickiewicz University, Poznań, 8 Uniwersytetu Poznańskiego Str., 61-614 Poznań, Poland; 2 Department of Chemistry, University of Milan, Via Golgi 19, 20133 Milan, Italy; 3 Faculty of Science and Technology, Jan Dlugosz University, Al. Armii Krajowej 13/15, 42-200 Czestochowa, Poland; 4 Department of Molecular Photophysics, Institute of Applied Physics and Mathematics, Gdańsk University of Technology, 11/12 Narutowicza Str., 80-233 Gdańsk, Poland; 5 Instituto di Scienze e Tecnologie Cimiche “Giulio Natta” – CNR, Via Fantoli 16/15, 20138 Milan, Italy; 6 School of Electrical Engineering and Robotics, Faculty of Engineering, Queensland University of Technology (QUT), Gardens Point, Brisbane, QLD 4001 Australia; 7 NanoBioMedical Centre, Adam Mickiewicz University, Poznań, 3 Wszechnicy Piastowskiej Str., 61-614 Poznań, Poland

**Keywords:** Electrochemistry, Energy, Photochemistry, Theoretical chemistry, Chemical physics, Quantum physics, Solar energy

## Abstract

The relatively low photon-to-current conversion efficiency of dye-sensitized solar cells is their major drawback limiting widespread application. Light harvesting, followed by a series of electron transfer processes, is the critical step in photocurrent generation. An in-depth understanding and fine optimization of those processes are crucial to enhance cell performance. In this work, we synthesize two new bi-ruthenium sensitizers with extended anchoring ligands to gain insight into underlying processes determining photovoltaic action mechanisms. The structure of the compounds has been confirmed, and their properties have been thoroughly examined by various techniques such as NMR, IR, elemental analysis UV–Vis, cyclic voltammetry, and electroabsorption. The experimental characterization has been supported and developed via extensive quantum-chemical calculations, giving a broad view of the presented molecules’ properties. Finally, the DSSC devices have been assembled utilizing obtained dyes. The photovoltaic and EIS measurements, combined with performed calculations and fundamental dyes characterization, unraveled an intramolecular electron transfer as an initial step of the electron injection process at the dye/semiconductor interface. The overall photovoltaic action mechanism has been discussed. Our study demonstrates the significance of the anchoring group architecture in the molecular design of new sensitizers for DSSC applications.

## Introduction

Dye-sensitized solar cells (DSSCs) have received great attention as efficient photovoltaic devices in the last decades due to their low cost and ease of fabrication. Even though the power-conversion-efficiency (PCE) of DSSCs has increased from ∼ 7 to ∼ 14%^1^, it is still a barrier to further penetration of DSSCs technology into the photovoltaic market. Improvement of electronic and optical properties of DSSCs materials is highly sought after to enhance the efficiency of DSSCs. The photoanode of DSSC comprises wide bandgap semiconducting metal oxides that do not absorb visible light. Commonly used methods to improve photoanodes include semiconductor film nanoarchitecture, doping, and interfacial engineering. Therefore, the metal-oxide surface can be sensitized by organic dyes.

**Figure 1:**
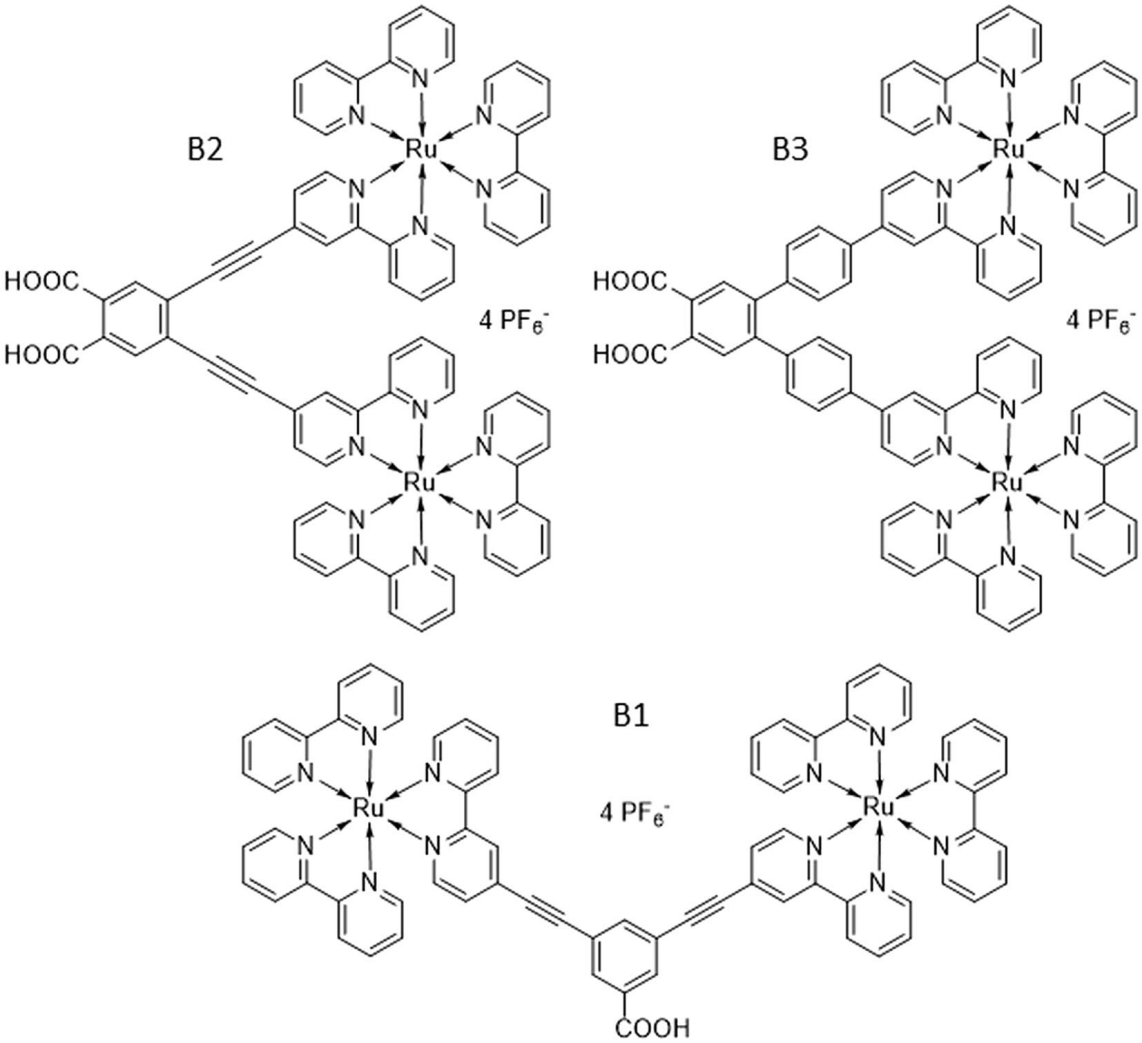
Structures of investigated dyes.

Various photosensitizers, including synthetic (organic and metal complexes) and natural dyes, have been investigated for DSSC applications^2–4^. The Ru-bipyridyl-based compounds are promising in photoconversion efficiency and operational stability. The mainstream research in ruthenium sensitizers is focused on structure modification of the ancillary ligands to improve their light-harvesting ability and electron transfer efficiency^5,6^. In our previous work^7,8^, a new approach to synthesizing the dinuclear tris(bipyridine)ruthenium(II) complexes was proposed (see Fig. 1). In that case, B1 dye molecules were anchored to the semiconductor through the π-expanded ligand, having a phenylene-ethylene moiety which increases the molar extinction coefficient^9^. Additionally, incorporating two Ru centers in one molecule may increase the generated charge concentration and electron injection via one active site on the semiconductor surface. Our previous investigations show a high extinction coefficient of the B1, indicating its potential as a sensitizing dye in DSSC. Unfortunately, the photovoltaic measurements show a low photon-to-current conversion ability of B1. Moreover, the measured dye loading parameter indicates that the adsorption of B1 on the TiO_2_ electrode is inefficient. The latter phenomenon may be related to the bulky molecule of B1 and/or the presence of only one anchoring –COOH group in its structure, which may decrease the binding abilities of this dye and the electron injection ability^5^.

In this work, elevating on the knowledge gained in our recent study on mononuclear tris(bipyridine)ruthenium(II) complexes^10^, we demonstrate the synthesis of other BX molecules (see Fig. 1). The new sensitizing dyes possess two anchor groups to improve their adsorption ability and binding strength, which are known factors for improving electron transport and injection in DSSCs^11^. Our previous findings^10^ also proved that the –COOH anchors position plays an important role in the electron injection process, so we decided to design new molecules with the anchors in the *para* position according to the Ru-centers. In the B3 molecule, the π-conjugated ethynyl ligand, present in molecules B1 and B2, is replaced with the phenyl group to stabilize the photogenerated charge and decrease recombination processes^12–14^. The main goal of this study is to provide further insight into the mechanism of charge transfer processes occurring in the dinuclear Ru complexes upon photoexcitation. Such an approach would lead to general conclusions allowing the design of more efficient dye molecules used in DSSCs.

## Results

### Electrochemical and photophysical properties

Cyclic voltammetry measurements have been performed to determine the electrochemical properties of investigated dyes. The obtained CV curves are shown in Fig. S1, while the corresponding electrochemical parameters are presented in Table S1. The CV data for the B1 dye obtained in our previous study^7,8^ are also referenced. In the anodic part of the CV curves, an intensive peak corresponding to one electron reversible redox process (Ru^2+^  → Ru^3+^  + e^−^) at the Ru centers of the molecules may be observed for all investigated dyes. The peak positions (E_pIa_) shift to the lower potential from 0.95 to 0.86 and 0.81 V for B1, B2, and B3 dyes, respectively. Such behavior is a consequence of differences in their HOMO energy level position caused by the change in the electronic structures of the molecules. The B1 molecule, with only one electron pulling –COOH anchor- has the highest oxidation peak potential. The additional –COOH anchor added to the B2 molecule downshifts the oxidation peak. The decrease of oxidation peak potential when the number of carboxylic groups increases was also reported by Shi et al.^15^ for the phthalocyanine zinc complexes with different numbers of –COOH moieties in the molecules. The substitution of the ethynyl linkers by phenyl ones in the B3 molecule downshifts further the oxidation potential. The shielding Ru centers from the anchoring –COOH groups by the phenyl linkers may explain such behavior of the B3 molecule, as observed earlier by Mary et al.^13^. A much more complex structure has been obtained at the reduction part of the CV patterns. The multistep reduction processes with a visible, sharp signal associated with adsorptive phenomena or sudden charge releases connected with structural rearrangements or unstable radical formation have been observed for all the investigated dyes. As in the case of the anodic part, a strong influence of the ligand structure on the first reduction peak position (E_pIc_) is visible. The additional anchoring group (–COOH) in the B2 molecule causes the slight shift of the E_pIc_ to the lower values compared to the value registered for B1, to − 1.67 from − 1.65 V, respectively. However, the shielding effect of phenyl linkers in the B3 structure is much stronger and shifts the B3 E_pIc_ peak to − 1.81 V. Such a substantial shift of the E_pIc_ value is a natural consequence of a LUMO level shift and may cause a decrease in the electron transfer ability between the donor (Ru) and acceptor (anchoring ligand). It is expected that the observed changes in the electrochemical properties of the dyes will affect the photovoltaic parameters of DSSCs.

The experimental UV–Vis absorption spectra of the B1, B2, and B3 molecules in acetonitrile (ACN) are presented in Fig. S2. The broad bands observed for all molecules with maxima at 460 nm correspond to the metal-to-ligand charge transfer (MLCT), characteristic of ruthenium polypyridine complexes^16,17^. Also, the absorption bands at 250 nm and those located at 280 nm correspond to the central ligands π–π* electron transfer (ILCT). The first MLCT peak of the B1 and B2 molecules, at 460 nm, is redshifted compared to the corresponding band registered for the B3 dye. The observed shift can be related to the modifications of the anchoring ligand structure. Nazeeruddin et al. ascribed such a shift to the presence of the electron-pooling carboxylic group in the molecules’ structure^18^. Brogdon’s group has observed a similar effect of spectral shift^11^ and has explained it by the charge transfer event strengthening via aromatic resonance stabilization of phenyl groups which decreases the electron-withdrawing effect of carboxyl groups or/and by the absence of steric hindrance in sensitizers investigated by them. As B3 dye has phenyl linkers in its structure, one may conclude that the replacement of phenyl by ethynyl linkers in the B1 and B2 dyes caused the redshift of the MLCT signal.

**Figure 2:**
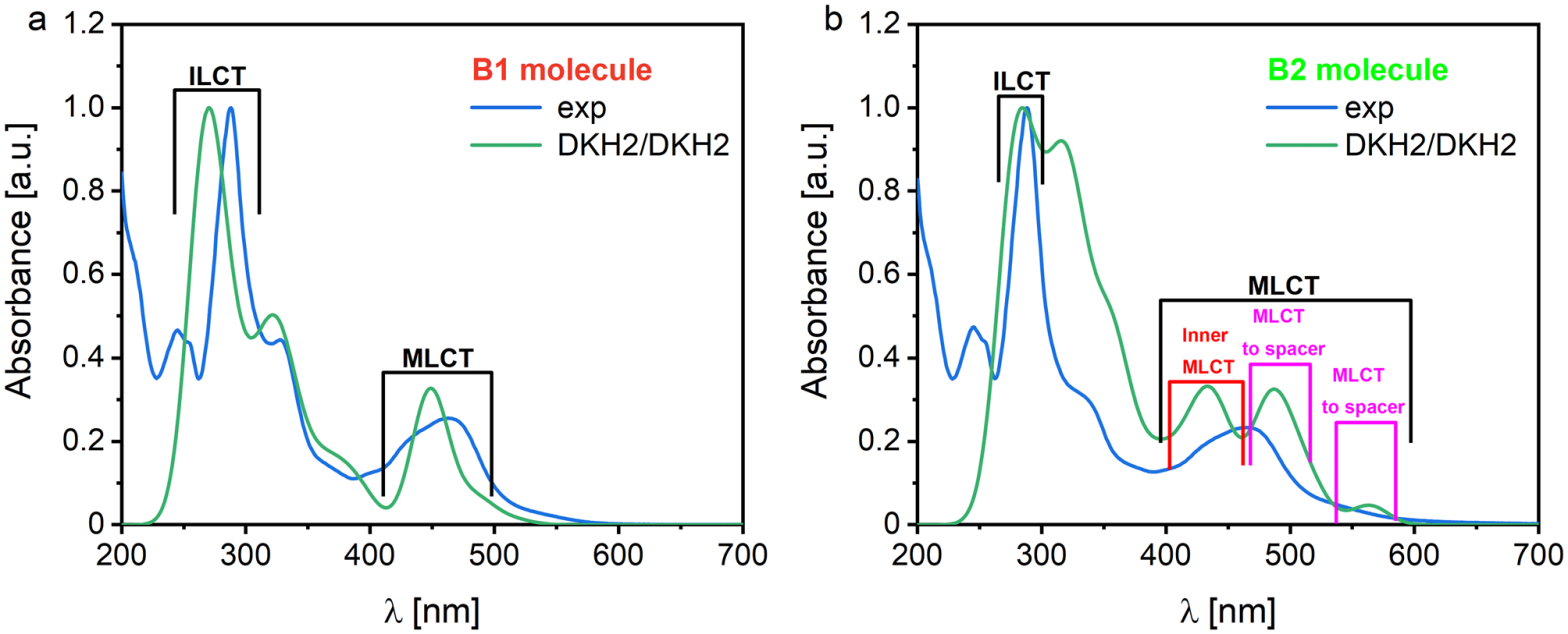
Normalized UV–Vis spectra for (**a**) B1 and (**b**) B2 measured experimentally in ACN (blue line) and calculated (green line) in a vacuum using BX structures optimized by the DFT/B3LYP-DKH2(jorge-TZP-DKH) and oscillators calculated by DFT/B3LYP-DKH2(jorge-TZP-DKH) method (DKH2/DKH2).

Our previous study^7,8^ concluded that an additional strong band present at 325 nm in the B1 molecule is associated with the more extended delocalized structure of the B1 anchoring ligand, acting as an optical antenna. The spectra predicted for the B1 and B2 molecules in a vacuum by quantum chemical calculations using the DKH2/DKH2 method are shown in Fig. 2. One can see that the performed calculations well reproduce the experimental data (see Fig. S2), especially the first MLCT peak and the ILCT peak for the B1 molecule. It is well done for the B1 molecule. The spectrum calculated for molecule B3 is similar to that calculated for B1. The first MLCT peak calculated for the B2 molecule is spread into two peaks. The calculations in a vacuum and an ACN were performed for the narrow range of spectrum responsible for the first MLCT transition to analyze more deeply the nature of the first MLCT peak responsible for the optical properties of the investigated molecules.

**Figure 3:**
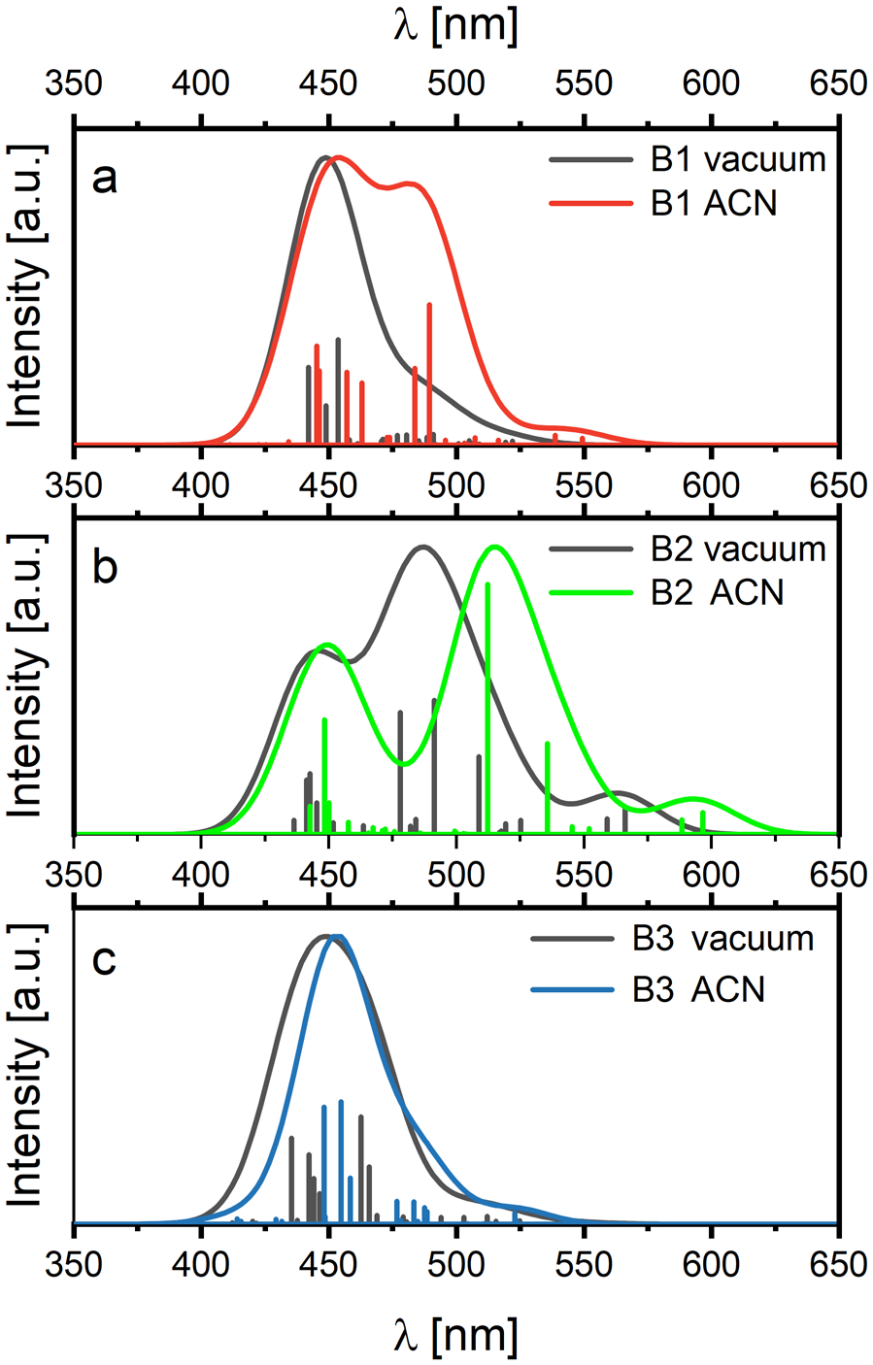
Normalized UV–Vis absorption spectra calculated by DKH2/DKH2 method for the B1, B2, and B3 molecules stabilized by the PF_6_^−^ in vacuum (black) and solvent ACN (red, green, blue, respectively).

The UV–Vis absorption spectra calculated for the B1, B2, and B3 molecules in vacuum using the DKH2/DKH2 method possess one peak centered at 450 nm (see Fig. 3). This peak is attributed to the MLCT transition observed for all molecules based on Ru(bpy)_3_^2+^ moiety^19^. For the B1 and B3 molecules, a small shoulder from the side of long waves in the absorption UV–Vis spectrum is seen. This peak is more significant for the B2 molecule. Analyzing the electron transitions responsible for the peaks laying around 480–490 nm, one can see that they are associated with the metal-to-ligand transition. However, the excited electrons are transferred to the orbitals located at the spacer. Also, the peak near 560 nm, significantly observed for the B2 molecule (see Figs. 2b, 3), is created by MLCT transition from Ru orbitals to the spacer-based orbitals. The peak at 480–490 nm is created by the transfer of electrons from HOMO-3 to LUMO (see Fig. S3), and the peak at 560 nm is associated with the HOMO to LUMO electron transition, but its oscillator strength is very low.

These transition states are also present in B1 and B3 molecules, but their oscillator strengths are very low (see Fig. 3). One can see that solvent enhance the peak intensity responsible for the MLCT transition based on metal-to-spacer transfer (480–490 nm) for B1 molecule. Under the solvent influence, the electrons transition from the metal to the spacer is still marked by very low oscillator strength for the B3 molecule. It can be explained by the fact that the phenyl group added to the spacer of the B3 molecules affects charge transfer from the donor to the acceptor moiety. The mentioned charge transfer is well seen for the B2 molecule. The solvent for all molecules does not affect the peak responsible for the metal-to-bipyridine transition.

The electron properties of the investigated molecules calculated by the DKH2/DKH2 method in a vacuum and ACN are presented in Table S2. The B1 and B3 molecules have very similar electric dipole moments, but molecule B2 possesses a much lower electric dipole moment. The dipole moment of all molecules increases in the ACN solvent. Most significantly, it is seen for the B2 molecule. The analysis of tested molecules’ electrical properties, especially dipole moments, argues that they will affect the semiconductor conducting band, changing the photovoltaic cells’ open-circuit voltage (V_OC_), as proved for the mono-ruthenium complexes^10^.

In Table S2, HOMO and LUMO energy values and the energy gap (E_g_) between them are calculated for B1, B2, and B3 molecules, and the data are compared to experimental results. Experimentally measured E_g_ has the lowest value for the B2. It allows us to conclude that the B2 will be characterized by the highest reactivity, which may result in the most effective adsorption of these molecules on the surface of the semiconductor from the BX series^20^. The HOMO energy of the investigated molecules is upshifted, going through B1 → B2 → B3. The higher the energy of the HOMO, the greater the capacity to donate electrons will be. It will have consequences in the electron injection in DSSC devices. The additional anchoring group downshifted the LUMO level of the B2 compared to the B1. The phenyl group of the spacer moiety in B3 upshifts the LUMO giving the highest value of all BX molecules.

**Figure 4:**
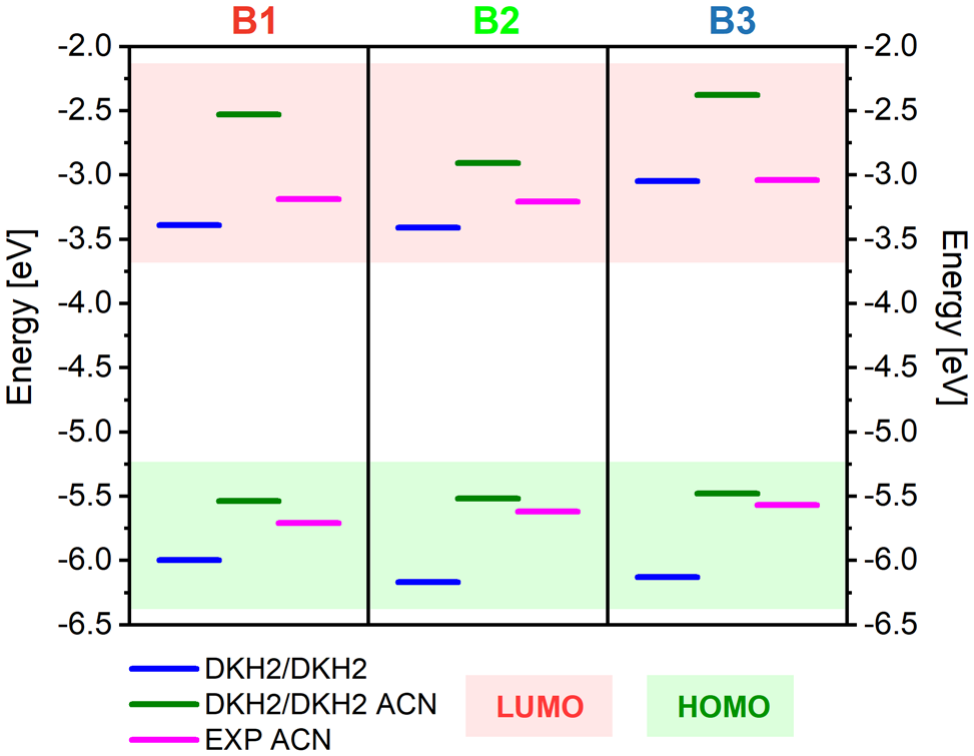
Energy diagrams calculated for B1, B2, and B3 molecules by DKH2/DKH2 in vacuum (blue line) and ACN (green line), and experimental data measured in ACN (violet line).

The frontier orbitals were calculated using the DKH2/DKH2 method in a vacuum and ACN. Comparing the HOMO and LUMO positions calculated for the BX molecules in a vacuum and ACN, one can see that the solvent shifts HOMO and LUMO into the higher energies for all molecules (see. Table S2, Fig. 4). The tendencies of the HOMO and LUMO energy changes obtained experimentally for the BX molecules are well reproduced by the calculations performed in ACN. However, discussing these results quantitatively, one can see that experimental HOMO energy is better reproduced by calculations performed in solvent, but the LUMO energies are in better agreement with the ones calculated in a vacuum. It means that the LUMO levels are only marginally affected by the environment, and it can be calculated correctly for the isolated molecule. The HOMO orbitals are more sensitive to the environment. The solvent does not change the E_g_ of the BX molecule significantly. It is also seen by analyzing the UV–Vis spectra presented in Fig. 3. It can be concluded that the significant increase in the energy of the LUMO of the B3 dye visible in the diagram compared to the B1 and B2 dyes is responsible for the hypsochromic shift of the MLCT band. This observation supports the above conclusion about the introduction of phenyl linkers into the B3 dye structure, which causes the π–π* and dπ–π* transitions to occur at higher energies. In addition, the HOMO levels are also shifted with each modification made, but not as significant as the LUMO levels.

The standard analysis, based on derivatives of absorption spectra and discrete parametrization of EA spectra in the form of molecular dipole moments using the Liptay formalism^21^, is difficult to reliably perform in spectral ranges with densely packed excited states. However, the EA spectra can be successfully compared with those computed by the DKH2/DKH2 method without imposing any restrictions on the shape of the EA signal spectral line^22^. The same approach can be applied to biruthenium complexes B1, B2, and B3, where much more excited states are involved in the EA signal. A reasonable compromise between the method’s accuracy and moderate computational cost for such large molecular systems is always worth considering. The EA curves for complex B1 calculated using the time-consuming DFT formalism (dashed line) and the faster semiempirical PM6 method (solid line) are compared in Fig. S4. In both cases, more than a hundred of the first singlet electronic transitions are included in the spectral range 18–34 kK. The abscissa of EA plots is expressed as a wavenumber in units of kilokaysers (1 kK = 10^3^ cm^−1^). The assumed bandwidth *w* = 1500 cm^−1^ is consistent with the dominant vibrational progression of 1300–1500 cm^−1^ energy spacing, equivalent to stretching modes of aromatic rings. Although the differences between the two curves can be easily recognized, the main spectral features are reproduced well by both methods, thus, the following EA results are presented using the PM6 method.

**Figure 5:**
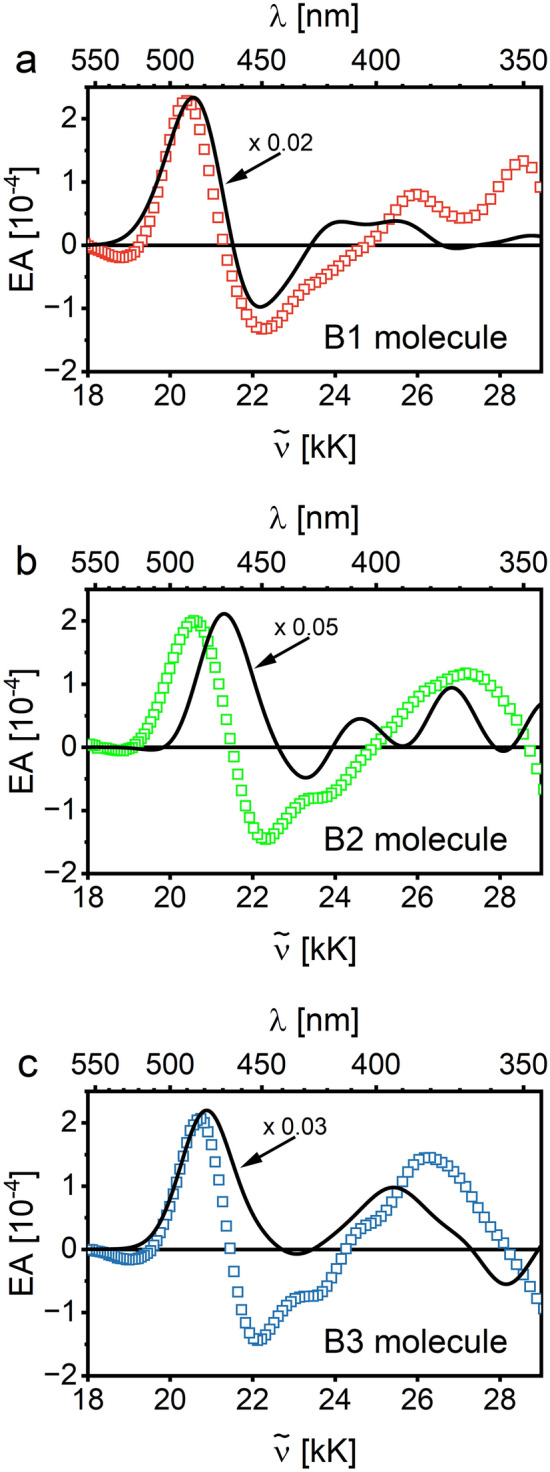
The experimental EA spectra of solid films of biruthenium complexes (squares) measured with the applied electric field ${F}_{\mathrm{rms}}=(6\pm 1)\times {10}^{5}\,\mathrm{V}/\mathrm{cm}$ in comparison with EA theoretical curves (solid lines) calculated for molecules in vacuum using the PM6 method with a bandwidth of *w* = 1.5 kK and the same electric field value, for complexes B1, B2, and B3.

In Fig. 5, experimental EA results for solid layers of biruthenium (B1, B2, and B3) complexes (squares) are compared with theoretical EA curves (solid lines) calculated for molecules in a vacuum, all related to an applied electric field of about 6 × 10^5^ V/cm. As can be seen, the spectral dependence of the EA signal in the 18–24 kK range, which includes the main low-energy absorption of MLCT character, is roughly represented by calculations based on the PM6 method. However, the predicted EA signal values for a molecule in a vacuum are about 20–50 times greater than the EA signals measured in solid films. These differences can undoubtedly be attributed to the breakdown in the solid state of both the *applied field approximation* used and the validity of the linear relationship with the concentration of the absorbance found in the Lambert–Beer formula (4). It is worth noting, however, that the approximation of the *Lorentz local field*, typical for solids, with the field correction factor $f=(\varepsilon +2)/3 \cong 1.7$(relative electric permittivity *ε* = 3) used here to convert results from vacuum to solid increases the observed signals' observed differences. Therefore, more realistic models of the local electric field that consider the nearest molecular neighbors should be developed in the future.

All mono- and biruthenium complexes of bipyridine, based on the [Ru(bpy)_3_]^2+^ ion, show a characteristic EA signal with a spectral shape similar to the absorption second derivative with a minimum of the negative lobe observed at about 22 kK. This common feature of EA was attributed to a highly delocalized and orbitally degenerate or quasi-degenerate excited state in [Ru(bpy)_3_]^2+^ based systems due to the robust nature of MLCT electronic states in such systems^22^. Based on Liptay formalism^21^ for a single electronic transition at about 22 kK in an isotropic system, the dipole moment change Δ*µ* (in debye unit, 1D = 3.34 × 10^–30^ C·m) upon this MLCT excitation can be evaluated according to the formula:

1 $$\Delta\mu =2.8\times {10}^{4} {\left(\frac{{3 EA}_{\mathrm{max}}}{ {D}_{0}}\right)}^\frac{1}{2}\frac{w}{{F}_{rms}} ,$$

where the amplitude *D*_0_ and the width *w* (expressed in cm^−1^) refer to the Gaussian band and the electroabsorption signal *EA*_*max*_, measured in an electric field with the rms value *F*_*rms*_ (in V/cm), refers to the minimum of EA spectrum observed at the exact wavenumber as the central position of the MLCT absorption band. Due to a congested manifold of electronic states in the considered spectral range, it is difficult to reliably determine the *D*_0_ value from the experimental absorption spectrum. Therefore, the value of the *D*_0_ parameter was evaluated in two limiting cases. Firstly, *D*_0_ = 0.04 was obtained for the Gaussian band at 22 kK derived from the global absorption spectrum deconvoluted into the sum of several Gaussian profiles, as shown in Ref.^22^ for a solid film of the B1 complex. The given *D*_0_ value corresponds to the linear absorption coefficient *α* = 1.5 × 10^4^ cm^−1^, which is a factor of 5 lower than the maximum value of this coefficient recorded in the appropriate spectral range. In the second case, it was assumed that the amplitude *D*_0_ equals the value of the total absorbance *D* at 22 kK in the global absorption spectrum of complexes B1, B2, or B3, respectively. Taking from the experiment, the average width of Gaussian bands, *w* = 2000 cm^−1^, the electric field intensity *F*_*rms*_ = 6 × 10^5^ V/cm and using the applied field approximation almost the same values of Δ*µ* = 10 ± 1 D (the first method) and Δ*µ* = 5 ± 1 D (the second method) for B1, B2, and B3 complexes were obtained, which is consistent with available literature data for another bipyridine Ru complexes^22–25^. It should be noted, however, that despite the observed similarity in the EA behavior of various Ru(II) bipyridine complexes, the above estimation of Δ*µ* should be treated with caution due to the somewhat complicated pattern of interaction of excited states with the electric field involved in the EA response.

### Photovoltaic parameters

**Figure 6:**
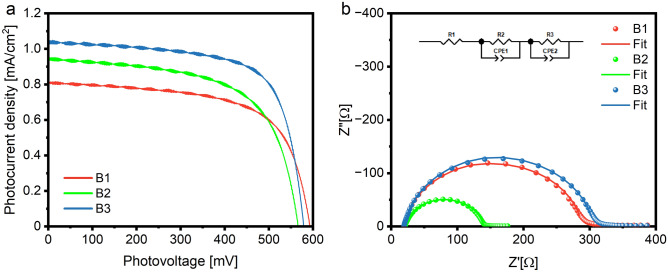
*I–V* curves registered for investigated cells (**a**), Nyquist plots registered for BX cells, and the equivalent circuit model used for fitting (**b**).

The *I–V* curves registered for investigated DSSCs are shown in Fig. 6a, while corresponding photovoltaic parameters are presented in Table 1. Obtained *V*_*OC*_ values vary from 579 for B3 dye to 593 mV for B1 dye. The open circuit voltage of DSSCs is usually defined as a difference between the Fermi level of a semiconductor and the Nernst potential of an electrolyte redox couple^26^, thus, it should not change when different sensitizers are used. The anchoring ligand may influence the *V*_*OC*_. A possible explanation of this process was proposed by Nazeruddin et al.^27^, a sensitizing molecule, during the adsorption process, transfers the protons to the semiconductor surface and generates the positive charge on the TiO_2_ surface. Such transfer generates a surface dipole that enhances a local electric field, increasing the dye adsorption yield. Simultaneously, the semiconductor surface protonation results in a downshift of the semiconductor CB, resulting in a *V*_*OC*_ decrease. The dye-loading (see *N*_*dye*_ parameter in Table 1) increases significantly for B2 and B3 dyes that have two –COOH anchoring groups when compared with *N*_*dye*_ for B1, which is probably caused by the more efficient protonation of the semiconductor surface and the CB shift that results in the decrease in the *V*_*OC*_^28^. The decreasing *V*_*OC*_ trend, simultaneous with the decrease of *µ* values, has also been observed by us in our previous work^10^. The *µ* values decrease in the following order B1 > B3 > B2 (see Table S2), analogous to the *V*_*OC*_ values. The above observation implies that the dipole moment of the dye molecule affects the CB position in the BX-sensitized cells.

**Table 1:** Photovoltaic parameters of DSSCs and dye loading values for investigated dyes.

Dye	*V*_*OC*_ (mV)	*J*_*SC*_ (mA/cm^2^)	*FF* (%)	*η* (%)	*N*_*dye*_ (nM/cm^2^)
B1	593	0.81	64.1	0.31	9.75
B2	566	0.95	62.0	0.33	15.9
B3	579	1.03	69.8	0.42	13.8

The photocurrent density (*J*_*SC*_) of investigated cells presents a slightly different trend than discussed *V*_*OC*_. The * J*_*SC*_ increases significantly with the addition of a second anchoring group into the molecule (in B2 dye) and with the substitution of ethynyl moieties for the phenyl ones (in B3 dye). Another factor that may affect the *J*_*SC*_ in DSSCs is the *N*_*dye*_ value. In general, the current density is increasing as the *N*_*dye*_ value increases^29^. The dye loading parameter significantly improves in double-anchor B2 and B3 dyes compared with single-anchor B1 (see Table 1). However, this dependence is not linear, suggesting that the discussed effect is not proportional, and other processes may affect the *J*_*SC*_^30^. It is worth noting that the increase of *J*_*SC*_ of B3 cell, when compared with B2, may also be associated with the increase of molar extinction coefficient at MLCT band growth (see ε_MLCT_ values in Table S1), suggesting more efficient photon harvesting by B3 dye. A similar relationship between ε and *J*_*SC*_ has been described by Yu et al.^31^. The additional –COOH anchor also modifies the LUMO level of the dyes (see Table S2). It was shown by Renall et al. that the decrease in LUMO level disturbs the electron injection process and may negatively influence *J*_*SC*_^32^. Here, it is observed that an additional anchor downshifts the LUMO level of B2 dye compared to B1. At the same time, the screening effect of phenyl linkers causes a significant increase in the LUMO level of B3 dye, reaching the highest value in the BX series. This considerable change in the LUMO level also improves the *J*_*SC*_ value of B3-sensitized cells. Moreover, our previous work proved that the *para* position of the anchor to the metallic center privileges the intramolecular charge transfer from Ru to the anchor compared with *meta* and *ortho* positions^10^. When considering Ru-centers’ position in the BX dyes (see Fig. 1), it may be concluded that in the B1 case, both donor moieties are in a less favorable *meta* position relative to the single-COOH anchor. On the other hand, incorporating the second anchor into the B2 and B3 molecules causes both Ru-centers to be in a *meta* position regarding the one anchor but simultaneously in a more favorable *para* position to the other anchor. Such structure should improve intramolecular charge transfer and, consequently, the electron injection process and* J*_*SC*_.

The parameter describing internal resistance and electron injection processes in solar cells is called fill factor (*FF*). It is a helpful tool to support the discussion about the electron transfer mechanism in DSSCs^33^. The lowest *FF* value has been registered for B2 cells, which suggests the highest resistance and/or efficient recombination processes in that device type. Electrochemical impedance spectroscopy (*EIS*) measurements have been performed to better understand the nature of *FF* changes, and the results are presented in Fig. 6b and Table S3. The serial resistance (*R*_1_) and the counter electrode resistance (*R*_2_), obtained by fitting Nyquist plots, registered for DSSCs with BX dyes do not vary much, which suggests good reproducibility of the device preparation process. Insignificant differences in the *R*_2_ values, especially registered for B2 cells, are probably caused by discontinuities or cracks in the Pt layer on the counter electrodes^34^. As the only part of the devices modified in this study was the sensitizing dye, the most important information may be extracted from the *R*_3_ value representing the resistance at the TiO_2_/dye/electrolyte interface. The lowest *R*_3_ value obtained for the B2 dye-sensitized devices supports our hypothesis that an additional –COOH anchor in the *para* position enhances the electron transfer in the TiO_2_-dye system. On the other hand, B2 devices showed the lowest *FF* value, which suggests, when considering simultaneous the lowest *R*_3_, a very effective recombination process across the entire DSSC device. The electron lifetime values, estimated from the maximum point of the *R*_3_ arc frequency, which is the lowest for the B2 devices, support the latter (see *τ* in Table S3). In the B3 dye case, when the phenyl linkers are incorporated into the molecule, the *R*_3_ resistance is even higher than registered for B1 devices. Still, thanks to the generated charge stabilization effect caused by phenyl linkers, the relatively long electron lifetime may be observed, while the *FF* value is the highest in the BX series. As a consequence of the abovementioned effects, the overall photon-to-current conversion efficiency (*η* value in Table S3) of B3 dye is the highest among the investigated devices.

**Figure 7:**
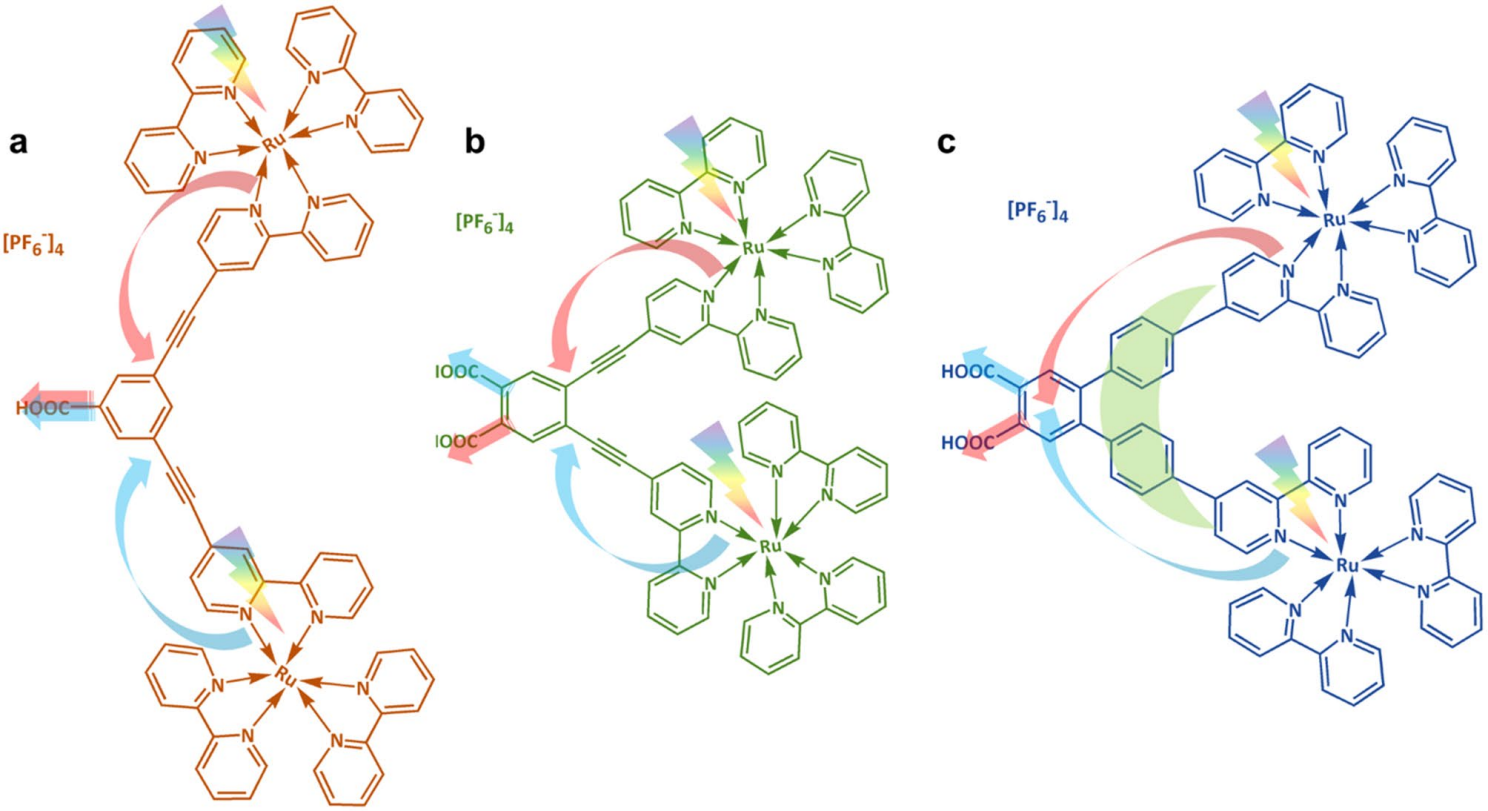
Proposed electron transport mechanism during the photovoltaic action in (**a**) B1, (**b**) B2, and (**c**) B3 sensitizers. The arrows’ colors indicate the possible route of intramolecular electron transfer from Ru centers to the anchors.

As a result of our study, we propose a probable electron transfer mechanism in BX dyes shown in Fig. 7. Single –COOH anchoring group in B1 dye seems to be a “bottleneck” for the electron injection process. Moreover, the less favorable *meta* position of this group (in relation to both Ru centers) additionally inhibits the intramolecular electron transfer from the Ru donor groups of the anchor. CV measurements indicate that in all the sensitizers studied, excitation of electrons upon photoexcitation coincides at both Ru^2+^ atoms, leading to a high charge concentration on the dye molecule. However, in the case of B1 dye, this process results in an ineffective energy dissipation rather than improving the electron injection process. Incorporating an additional anchor, located at the more electron transfer effective *para* position, significantly improves the electron injection process in the B2 molecule (lower *R*_3_ value). Unfortunately, the decrease in LUMO energy level, which suppresses the electron injection forces, in combination with the relatively low *V*_*OC*_ value, mainly due to the high dipole moment of the B2 molecule, supports recombination processes decreasing the electron lifetime and results in only a marginal increase in the efficiency B2 cells. Finally, the phenyl linkers incorporated in the B3 molecule stabilize excited electrons and mitigate rapid electron transfer processes in the B2 molecule, increasing the *R*_3_ value in the B3 molecule, extending the electron lifetime, and effectively reducing recombination. Simultaneously, a downshift of the B3 LUMO level supports electron transfer forces that lead to * J*_*SC*_ enhancement and improve the overall photovoltaic action efficiency of B3-sensitized devices.

## Discussion

In summary, we have demonstrated an effective synthesis route of two new ruthenium complexes, with two –COOH anchor groups located in *meta* and in *para* position with regards to each of the metal centers, followed by the comprehensive experimental and computational study of the electronic processes determining the final performance of DSSCs. The analysis of the photocurrent–voltage curves shows that introducing the second anchor group to the ruthenium complex and changing its position in the aromatic ring from the *meta* to the *para* one increases the efficiency of the DSSC. Moreover, the replacement of the ethynyl linker by a phenyl linker led to an upshift in the LUMO level of the B3 dye, which increased the driving forces of electron injection into the TiO_2_ conduction band. Electrochemical impedance spectroscopy showed that the additional anchoring group significantly improved the charge transport (in the B2 dye concerning B1), while the short lifetime of the injected electron in the B2 system indicates a rapid recombination process. In the B3 case, even though the shielding properties of phenyl connectors have caused an increase in the cell's internal resistance, this system turned out to be the most effective out of the entire BX series, primarily due to the extended electron lifetime. Light-harvesting abilities of the B3 molecule have been enhanced after introducing phenyl groups, which increased the generated photocurrent density despite the less efficient adsorption of B3 on the electrode surface. The low photoconversion efficiency of B2 may also be attributed to the aggregation of dye molecules on the photoelectrodes surface, confirmed by computer simulations. Based on our findings, we propose an intramolecular electron transfer mechanism preceding the electron injection process at the dye-semiconductor interface. The intramolecular transfer depends on the anchoring ligand structure, especially on the number and position of anchors and the extension of the π-electrons structure. The presented results are crucial for further developing new sensitizers to ensure the highest possible DSSCs photoconversion efficiency.

## Methods

### Synthetic procedures

The detailed synthetic procedures performed in this work have been described in the Supplementary Information file. 4-Bromo-2,2ʹ-bipyridine was synthesized according to the procedure described by us earlier^35^, and 4,5-dibromophthalic acid was obtained according to the old procedure described by Zincke and Fries^36^ that gives higher product yields and less laborious procedure than methods based on 4,5-dibromo-o-xylene oxidation.

The NMR spectra were recorded on Bruker Avance II 400 MHz and Agilent DD2 800 MHz at 297 K, using tetramethylsilane as the internal standard. All NMR spectra recorded are shown in Figs. S5–S31 in the Supplementary Information file of this paper. Elemental analyses were made on Vario ELIII elemental analyzer. Melting points were determined on the Boethius apparatus (Boethius HMK, Germany) and were uncorrected.

### Details of quantum chemical calculations

The geometries of the B1, B2, and B3 molecules were found by applying the total energy minimization procedure in a vacuum using the ab initio methodology based on the Hartree–Fock (HF) formalism available in the Gaussian 16 program package^37^. The mentioned procedure was performed for molecules in the hexafluorophosphate (PF_6_^−^) environment. Four PF_6_^−^ molecules were added to each simulated BX molecule as stabilizers. Quantum chemical calculations were carried out applying the mix basis set. The LanL2DZ basis set with an effective core potential was used for the Ru atom, and the 6-31G basis set for the remaining atoms. The molecular structures were optimized with the gradient convergence tolerance of less than 10^–5^ Hartree/Bohr at a restricted Hartree–Fock (RHF) level^38^. Equilibrated geometries of the molecules were found by applying the quadratic approximation (QA) optimization algorithm based on the augmented Hessian technique. At the end of the geometry search, the Hessian evaluation was performed to exclude structures giving the negative modes and ensure a thermodynamic equilibrium of the molecule. Then, the geometries of the BX molecules were optimized by applying the DFT/B3LYP method augmented by the 2nd-order scalar relativistic effects within the Douglas–Kroll–Hess (DKH2) formalism^39–41^ using the jorge-TZP-DKH basis set^42^. In our previous work, it was proved that the geometry of the tris(bipyridine)ruthenium(II) based molecules optimized by DFT/B3LYP-DKH2 (jorge-TZP-DKH) could reproduce the experimental results^22^, but the calculations are very time-consuming.

The electron and optical properties of the studied BX molecules were calculated for their structure optimized by the DFT/B3LYP-DKH2 (jorge-TZP-DKH) method using also the DFT/B3LYP-DKH2(jorge-TZP-DKH) formalism. This method is designated in the text as DKH2/DKH2. These calculations were performed using the Gaussian 16 program package. The calculations were performed for molecules in the PF_6_^−^ environment. The RHF SCF energy convergence criterion was chosen to be 10^–12^ Hartree. The UV–Vis absorption spectra were calculated using the iterative Davidson algorithm with an accuracy of 10^–12^ Hartree. The UV–Vis spectra were also calculated for the molecular geometries optimized by HF formalism, described above, using the semiempirical parametrized PM6 method^43^. In this case, all computational parameters were selected as in the DKH2/DKH2 calculations.

The ACN solvent effects were also taken into account using the conductor-like Polarizable Continuum Model (C-PCM)^44,45^. The solvent radii and dielectric constants were assumed to be the same as the parameters collected in the Gaussian code. Electron and optical properties of the BX molecules in ACN solvent were calculated using DKH2/DKH2 formalism with the parameters described above.

### Electrochemistry and spectroscopy

Redox properties and HOMO and LUMO energy levels were investigated by cyclic voltammetry (CV) using a 5 × 10^–4^ M solution of BX dyes in 0.1 M tetrabutylammonium hexafluorophosphate, acting as supporting electrolyte, in acetonitrile. A 25 ml measuring solution was poured into a 50 ml conical shape electrochemical vessel and bubbled with 5.0 N Ar for deaeration. The measurements were performed in a three-electrode setup with a 0.0314 cm^2^ glassy carbon (GC) working electrode, a Pt spring counter electrode, and Pt wire as a pseudoreference electrode. The working electrode potential was normalized against Fc/Fc^+^ redox couple as the intersolvental standard recommended by IUPAC^46^. The GC electrode surface was restored before every measurement series and, whenever necessary, by mechanical polishing with artificial diamond powder on the wet polishing cloth. The CV curves have been registered on a Gamry Interface 1010 E potentiostat–galvanostat.

UV–Vis absorption spectra were obtained on a Shimadzu UV-3600 Plus UV–Vis–NIR Spectrophotometer in a 1 cm path-length quartz cell using 10^–5^ M acetonitrile solutions of investigated dyes.

### Electroabsorption measurements

The electroabsorption (EA) was investigated using a phase-sensitive technique in a sandwich cell arrangement, quartz substrate/Al/Ru-dye film/Al, with two vacuum-evaporated semi-transparent Al electrodes and a beam of light passing perpendicularly through the sample. Solid, neat films of 40–50 nm thickness for Ru dyes (B1, B2, and B3) were prepared by a spin coating method using acetonitrile as solvent. For samples placed in a modulating sinusoidal electric field (ω), the EA signals are defined by:

2 $$EA=\frac{{I}_{2\omega }}{{I}_{0\omega }} ,$$

where *I*_*2ω*_ is the rms value of the (2ω)-Fourier component of the transmitted light beam intensity probed by a lock-in amplifier (PAR, model 5210) and *I*_*0ω*_ is the value of the (0ω)-Fourier component measured with an electrometer (Keithley, model 2000). Further details of the experimental procedures can be found elsewhere^22^.

Theoretical EA spectra based on the DFT/B3LYP-DKH2(jorge-TZP-DKH) or PM6 numerical simulations were determined by subtracting the absorption spectra calculated without an external electric field from the spectra calculated in the presence of an electric field. Absorption spectra for electric fields directed along the molecule’s principal axes (-x, x, -y, y, -z, z) were averaged for the isotropic distribution of molecular orientations. The absorption spectra were composed ($D=\sum_{n}{D}_{n}$) from Gaussian profiles,

3 $${D}_{n}={D}_{0n} \mathrm{exp}\left[-4 \mathrm{ln}2 {\left(E-{E}_{n}\right)}^{2 }/ {{w}_{n}}^{2} \right] ,$$

where *w*_*n*_ = FWHM (full width at half maximum), *E*_*n*_ is the calculated energy position, and *D*_0*n*_ the maximum intensity (height) of the Gaussian band assigned to the *n*-th electronic transition. To compare the theoretical results of oscillator strength *f*_*n*_ calculated in a vacuum with the experimental data obtained for solid films, the absorbance of the Gaussian band for the *n*-th electronic transition was calculated assuming the validity of the Lambert–Beer law:

4 $${D}_{0\mathrm{n}}=c l \frac{{f}_{\mathrm{n}}}{4.6\times {10}^{-9}{ w}_{n}} ,$$

where *c* is the molar concentration (in M), *l* is the layer thickness (in cm), and *w*_*n*_ is the bandwidth (in cm^−1^). Typically, the same width, *w*_*n*_ = *w*, was arbitrarily assumed for all bands involved. For the molar concentration, *c* = 2.4 M was estimated, which correlates well with the values obtained from the crystal structures of bipyridine ruthenium [Ru(bpy)_3_]^2+^ and phenyl-pyridine iridium Ir(ppy)_3_ complexes^47,48^.

### Photovoltaic performance

The photovoltaic devices have been prepared by applying our standard methods published elsewhere^49,50^. In brief, working electrodes have been prepared by applying P25 titania paste (prepared by mixing 3 g P25, 1 ﻿ml acetic acid, 37 ml ethanol, 10 ml terpineol, and 1.5 g ethyl cellulose and evaporating an excess amount of ethanol on a rotary evaporator) on FTO substrates using the *doctor-blade* technique. After annealing at 450 °C, substrates were cooled down, treated with 40 mM TiCl_4_ water solution for 1 h at 70 °C, dried, and annealed again at 450 °C. As prepared, electrodes were immersed in 10^–4^ M acetonitrile solution of investigated dyes and kept in the dark at ambient temperature overnight. Subsequently, working electrodes were washed, dried, and assembled in the sandwich device with Pt/FTO counter electrodes, using 25 µm thick hot-melt ionomeric foil as a sealant and separator. Liquid electrolyte (containing 0.03 M iodine, 0.6 M 1-propyl-3-methylimidazolium iodide, 0.1 M guanidine thiocyanate, and 0.5 M 4-tertbutylpiridine in acetonitrile) have been injected into the cells through two 0.8 mm diameter holes predrilled in the counter electrodes. The final sealing of the cells was done using two pieces of ionomeric foil and a microscope cover slide. The cells’ active area was approximately 0.125 cm^2^, and the titania layer thickness was about 11 µm; no shading mask was used for photovoltaic measurements. Four cells were prepared for each presented dye, and the results shown are for the best ones; however, the photovoltaic parameters of all prepared cells are collected in Table S4. To estimate the amount of dye adsorbed on the working electrodes, the approx 3 cm^2^ electrodes were prepared. After immersion in the sensitizer solution, dye molecules were immersed in 2 M ammonia solution in acetonitrile for 30 min to desorb the dye. The obtained desorbed dye solution has been examined using the UV–Vis technique on Varian Cary 50 spectrometer, using the calibration curve method.

Photovoltaic parameters (*I–V *curves) measurements have been performed under AM 1.5G illumination using Abet Sun 2000 solar simulator. The light intensity was calibrated to 1 sun using a ReRa Systems reference cell with a KG5 filter. Electrochemical impedance spectroscopy (*EIS*) has been performed in the dark over a frequency range from 0.1 Hz to 100 kHz at * V*_*OC*_ bias potential, with sinusoidal* V*_*AC*_ = 10 mV. The I–V curves and * EIS *spectra have been registered on a Gamry Interface 1010 E potentiostat–galvanostat. * EIS *data have been fitted using ZView 3.2 software.

### Supplementary Information

Supplementary Information.

## Data Availability

All the data are available on request from the corresponding author.
