# Supplementary material for: The importance of anchoring ligands of binuclear sensitizers on electron transfer processes and photovoltaic action in dye-sensitized solar cells
Source: Sci Rep. 2023 Oct 5;13:16808. doi: 10.1038/s41598-023-44015-8 (PMC10556067; doi:10.1038/s41598-023-44015-8)
Supplement: Supplementary file 1 — Supplementary Information. [file 41598_2023_44015_MOESM1_ESM.docx]

The importance of anchoring ligands of binuclear sensitizers on electron transfer processes and photovoltaic action in dye-sensitized solar cells.

Aleksandra Bartkowiak^1,2^, Oleksandr Korolevych^3^, Błażej Gierczyk^1^, Daniel Pelczarski^4^, Alberto Bossi^5^, Maciej Klein^6^, Łukasz Popenda^7^, Waldemar Stampor^4^, Małgorzata Makowska-Janusik^3^, Maciej Zalas^1*^

^1^ Faculty of Chemistry, Adam Mickiewicz University, Poznań, 8 Uniwersytetu Poznańskiego str., 61-614 Poznań, Poland;

^2^ Department of Chemistry, University of Milan, Via Golgi 19, 20133 Milano, Italy;

^3^ Faculty of Science and Technology, Jan Długosz University, Al. Armii Krajowej 13/15, 42-200 Częstochowa, Poland;

^4^ Department of Molecular Photophysics, Institute of Applied Physics and Mathematics, Gdańsk University of Technology, 11/12 Narutowicza str., 80-233 Gdańsk, Poland;

^5^ Istituto di Scienze e Tecnologie Molecolari – CNR, via Fantoli 16/15, 20138 Milano, Italy;

^6^ School of Electrical Engineering and Robotics, Faculty of Engineering, Queensland University of Technology (QUT), 2 George Street, Brisbane, Queensland 4000, Australia;

^7^ NanoBioMedical Centre, Adam Mickiewicz University, Poznań, 3 Wszechnicy Piastowskiej str., 61-614 Poznań, Poland;

* corresponding author e-mail address: maciej.zalas@amu.edu.pl

**Supplementary materials**

|  |
| --- |
| Figure S1. CV curves registered for B1 (a)^1,2^, B2 (b), and B3 (c) dyes at 0.2 V/s. |

| Table S1. | Electrochemical parameters of investigated dyes determined or calculated from CV experiments | | | | | | |
| --- | --- | --- | --- | --- | --- | --- | --- |
| Dye | E_pIIIc_ (V) | E_pIIc_ (V) | E_pIc_ (V) | E^0^_Ic_ (V) | E_pIa_ (V) | E^0^_Ia_ (V) | ε_MLCT_ (10^4^ M^-1^cm^-1^) |
| B1^1,2^ | -2.23 | -1.85 | -1.65 | -1.61 | 0.95 | 0.91 | 4.13 |
| B2 | -2.28 | -1.86 | -1.67 | -1.59 | 0.86 | 0.82 | 3.68 |
| B3 | -2.30 | -2.00 | -1.81 | -1.77 | 0.81 | 0.77 | 4.22 |

|  |
| --- |
| Figure S2. UV-vis absorption spectra of B_1_, B_2_, and B_3_ molecules measured experimentally in ACN. |

| 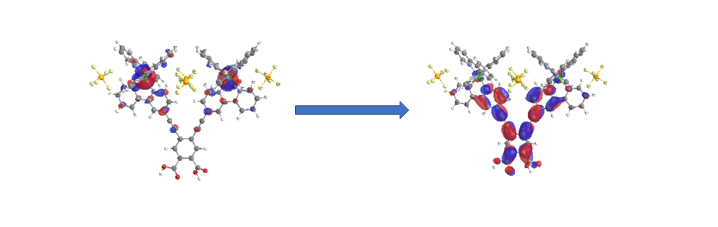 |
| --- |
| Figure S3. Schematic representation of the metal-to-spacer transfer (480-490 nm) representing first MLCT transition calculated for B2 molecule by DKH2/DKH2 method in a vacuum. |

| Table S2. | Electric dipole moments, the HOMO-LUMO level values, and the values of the energy gap (E_g_) between them, obtained from the theoretical calculations of DKH2/DKH2 (in a vacuum and ACN medium) and experimental data obtained using cyclic voltammetry (in ACN) for B1, B2, and B3 molecules | | | | | |
| --- | --- | --- | --- | --- | --- | --- |
|  | environment | µ (D) | calculated | | experimental | |
|  |  |  | HOMO (eV) | LUMO (eV) | HOMO (eV) | LUMO (eV) |
| B1 | vacuum | 5.66 | -6.00 | -3.39 | - | - |
|  |  |  | E_g_ = 2.61 eV | |  |  |
|  | ACN | 6.23 | -5.54 | -3.41 | -5.71 | -3.19 |
|  |  |  | E_g_ = 3.01 eV | | E_g_ = 2.52 eV | |
| B2 | vacuum | 3.46 | -6.17 | -3.41 | - | - |
|  |  |  | E_g_ = 2.76 eV | |  |  |
|  | ACN | 5.60 | -5.52 | -2.91 | -5.62 | -3.21 |
|  |  |  | E_g_ = 2.61 eV | | E_g_ = 2.41 eV | |
| B3 | vacuum | 5.29 | -6.13 | -3.05 | - | - |
|  |  |  | E_g_ =3.08 eV | |  |  |
|  | ACN | 7.43 | -5.48 | -2.38 | -5.57 | -3.04 |
|  |  |  | E_g_ =3.10 eV | | E_g_ =2.53 eV | |
| *values calculated from the E^0^_Ic_ and E^0^_Ia_ parameters obtained in CV experiments (see Table S1.) | | | | | | |

| Table S3. | Resistance parameters and electron lifetime registered and calculated from EIS measurements | | | | |
| --- | --- | --- | --- | --- | --- |
| Dye | | R_1_ (Ω) | R_2_ (Ω) | R_3_ (Ω) | τ (ms) |
| B1 | | 19.7 | 7.7 | 261.3 | 0.79 |
| B2 | | 21.3 | 2.9 | 115.7 | 0.32 |
| B3 | | 19.5 | 10.2 | 276.7 | 0.79 |

| Table S4 | The PV parameters obtained for all investigated cells | | | | |
| --- | --- | --- | --- | --- | --- |
| Cell | | V_OC_ [mV] | J_SC_ [mA/cm^2^] | FF [%] | η [%] |
| B1_1* | | 593 | 0.81 | 64.1 | 0.31 |
| B1_2 | | 585 | 0.79 | 63.1 | 0.29 |
| B1_3 | | 577 | 0.79 | 62.9 | 0.29 |
| B1_4 | | 594 | 0.78 | 62.2 | 0.29 |
| B2_1* | | 566 | 0.95 | 62.0 | 0.33 |
| B2_2 | | 553 | 0.93 | 57.4 | 0.30 |
| B2_3 | | 563 | 0.89 | 60.0 | 0.30 |
| B2_4 | | 569 | 0.92 | 60.6 | 0.32 |
| B3_1 | | 581 | 0.88 | 66.9 | 0.34 |
| B3_2 | | 561 | 1.02 | 69.6 | 0.40 |
| B3_3* | | 579 | 1.03 | 69.7 | 0.42 |
| B3_4 | | 597 | 0.88 | 67.7 | 0.36 |
| * Result taken to the main text (best for each dye). | | | | | |

| 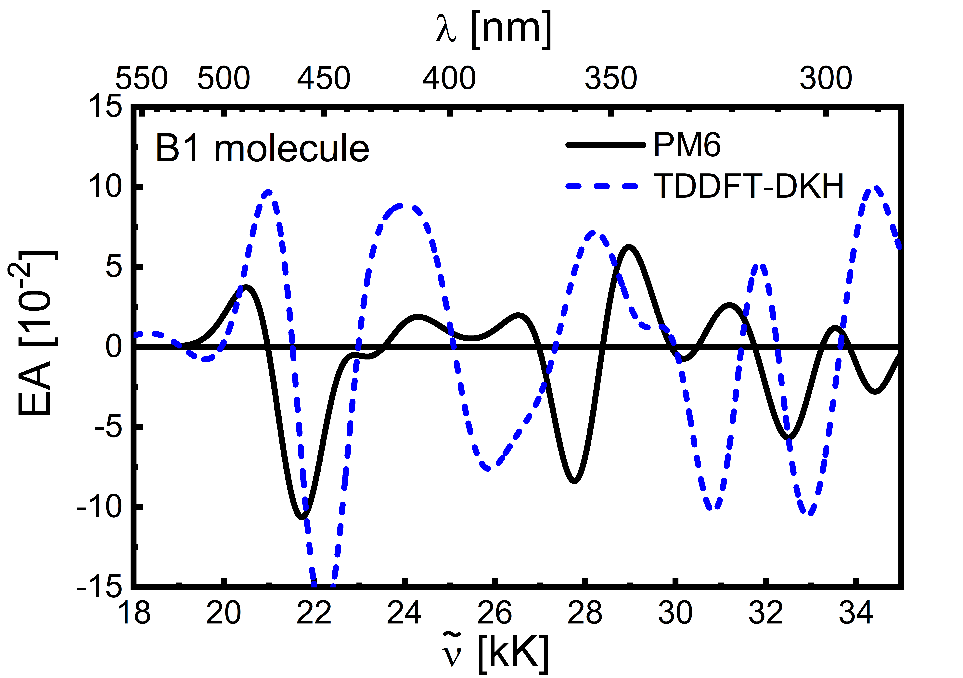 | |
| --- | --- |
|  | |
| Figure S4. Comparison of theoretical EA spectra calculated for the B1 molecule using the PM6 (solid line) and TDDFT-DKH (dashed line) methods for the electric field strength of 10^-3^ a.u. (1 a.u. = 5.14·10^9^ V/cm), assuming a bandwidth of 1.5 kK for all electronic transitions. | |
|  |  |
|  |  |

**Synthetic procedures**

| 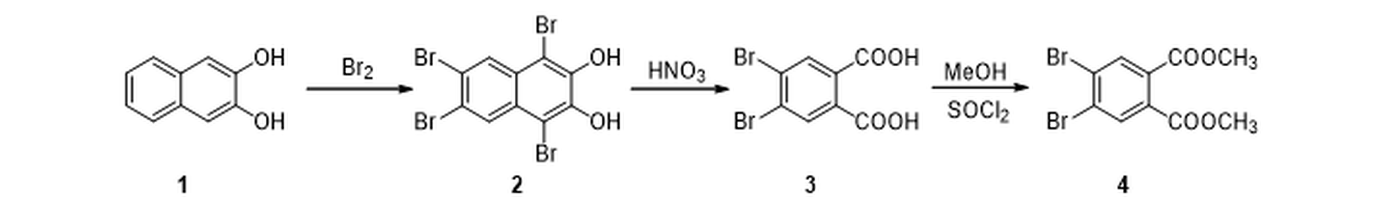 |
| --- |
| Scheme S1. Synthesis of the compounds **2**-**4**. |

**1,4,6,7-Tetrabromo-2,3-dihydroxynaphthalene (2)**

2,3-Dihydroxynaphthalene (**1**; 10.00 g, 62.4 mmol) was dissolved in anhydrous acetic acid (100 mL). The stirred solution of **1** bromine (16.5 mL, 0.322 mol) was added dropwise. The mixture obtained was heated under reflux for 5 h and cooled. The crystals formed were filtered off and crystallized from chloroform. The product was obtained as off-white crystals, yield 27.3 g (92%), m.p. 240-241°C.

^1^H NMR ([^2^H]_6_-acetone): δ 8.34 (s, 2H; **H**2), ca. 2.0 (bs, 2H; O**H**).

^13^C NMR ([^2^H]_6_-acetone): δ 146.4 (**C**5), 131.1 (**C**2), 128.7 (**C**3), 122.1 (**C**1), 104.4 (**C**4).

**4,5-Dibromophthalic acid (3)**

20 g of **2** (42 mmol) was heated with 400 mL of nitric acid (65% v/v) under reflux over ca. 5 h until the nitric oxides stopped being produced. After cooling, the crystals formed were filtered off and recrystallized from water. The acid was obtained as colorless needles, yield 12.20 g (90%), m.p. 207-210°C (with dehydration).

^1^H NMR ([^2^H]_6_-acetone): δ 8.07 (s, 2H; **H**2), 3.8 (bs, 2H; COO**H**).

^13^C NMR ([^2^H]_6_-acetone): δ 166.7 (**C**OOH), 134.8 (**C**2), 134.2 (**C**3), 128.0 (**C**1).

**Dimethyl 4,5-dibromophthalate (4)**

12.00 g of **3** (37 mmol) was dissolved in dry methanol (250 ml) and to this solution, thionyl chloride (20 ml) was added dropwise. The mixture was stirred over 2 h and the solvent was evaporated. The residue was dissolved in dichloromethane (200 ml), washed with 5% aqueous NaHCO_3_, the organic layer was dried over Na_2_SO_4_ and the solvent was evaporated. The product obtained as a white solid was used without further purification. Yield: 12.37 g (95%), m.p. 82-83°C.

^1^H NMR ([^2^H]-chloroform): δ 7.96 (s, 2H; **H**2), 3.91 (s, 6H; OC**H**_3_).

^13^C NMR ([^2^H]-chloroform): δ 166.0 (**C**OOH), 133.9 (**C**2), 131.9 (**C**1), 128.2 (**C**3), 53.0 (O**C**H_3_).

The protected boronic acid (**9**) was obtained according to the method reported by Welter et al.^4^.The 4-(4-bromophenyl)-2,2’-bipyridine was prepared using the method published by Cordaro et al.^5^.

**3-(4-Bromophenyl)-1-(pirydyn-2-yl)-2-propen-1-one (6)**

To the solution of 5.00 g of 4-bromobenzaldehyde (27 mmol), 3.63 g of 2-acetylpyridine (30 mmol) in methanol (100 mL), 25 mL of an aqueous solution of NaOH (1 M) was added, and the suspension obtained was stirred over 4 h. After then, the mixture was cooled in an ice bath and the precipitate formed was filtered off. After recrystallization from methanol, **6** was obtained as yellow crystals. Yield: 5.68 g (73%), m.p. 99-101°C.

The product is a mixture of *cis* and *trans* isomers (ca. 0.15:1). NMR data for *trans* isomer are reported.

^1^H NMR ([^2^H]-chloroform): δ 8.74 (ddd, 1H, 1.1, 1.7 & 4.7 Hz; **H**5), 8.30 (d, 1H, 16.1 Hz; **H**7), 8.19 (dt, 1H, 1.1 & 7.6 Hz; **H**2), 7.88 (td, 1H, 1.7 & 7.6 Hz; **H**3), 7.85 (d, 1H, 16.1 Hz; **H**8), 7.59 (*pseudo*d, 2H; **H**10), 7.54 (*pseudo*d, 2H; **H**11), 7.49 (ddd, 1H, 1.1, 4.7 & 7.6 Hz; **H**4).

^13^C NMR ([^2^H]-chloroform): δ 189.3 (**C**6), 154.1 (**C**1), 148.9 (**C**5), 143.2 (**C**8), 137.1 (**C**3), 134.1 (**C**9), 132.1 (**C**11), 130.1 (**C**10), 127.0 (**C**4), 124.8 (**C**12), 123.0 (**C**7), 121.5 (**C**2).

| 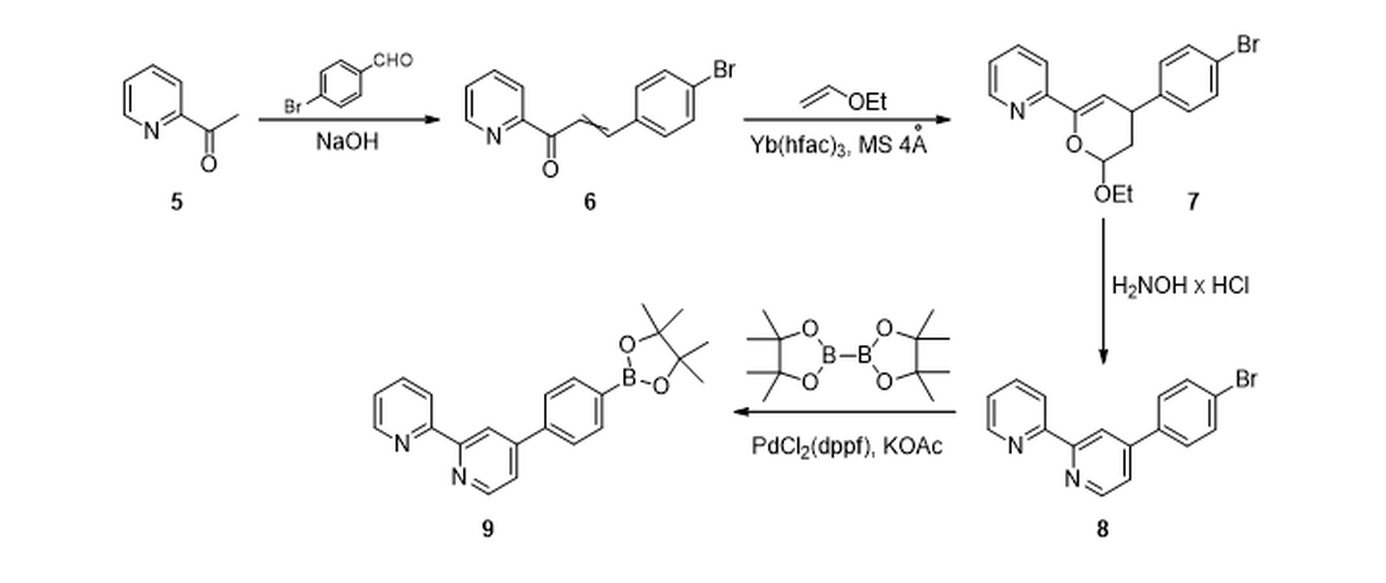 |
| --- |
| Scheme S2. Synthesis of the compounds **6** - **9**. |

**4-(4-Bromophenyl)-2-ethoxy-6-(pirydyn-2-yl)-3,4-dihydro-2*H*-pyrane (7)**

5.60 g of **6** (19.4 mmol) was dissolved in dry, freshly distilled THF (100 mL). To this solution, 0.5 g of ytterbium tris(hexafluoroacetylacetonate) dihydrate (Yb(hfac)_3_; 3.4 mol%), 20 mL (15.06 g, 0.209 mol) of ethyl vinyl ether and 6 g of freshly calcinated 4Å molecular sieves was added. The mixture was stirred over 48 h at room temperature under argon. Then the solids were filtered off and the solvent was evaporated. The product was purified by column chromatography (SiO_2_, dichloromethane/hexane 1:1 v/v) and obtained as a colorless oil. Yield: 6.08 g (87%).

^1^H NMR ([^2^H]-chloroform): δ 8.55 (dd, 1H, 1.1, 1.8 & 4.8 Hz; **H**5), 7.70 (td, 1H, 1.8 & 7.7 Hz; **H**3), 7.65 (dt, 1H, 1.1 & 7.7 Hz; **H**2), 7.41 (*pseudo*d, 2H; **H**11), 7.20 (ddd, 1H, 1.1, 4.8 & 7.7 Hz; **H**4), 7.18 (*pseudo*d, 2H; **H**10), 6.16 (dd, 1H, 1.2 & 2.8 Hz; **H**7), 5.26 (dd, 1H, 2.1 & 8.4 Hz; **H**14), 4.08 (dq, 1H, 7.0 & 9.5 Hz; **H**15), 3.79 (ddd, 1H, 2.8, 6.9 & 9.9 Hz; **H**8), 3.71 (dq, 1H, 7.1 & 9.5 Hz; **H**15), 2.36 (dddd, 1H, 1.2, 2.1, 6.9, 13.3 Hz; **H**13), 1.97 (ddd, 1H, 8.4, 9.9, 13.3 Hz; **H**13), 1.28 (t, 3H, 7.1 Hz; **H**16).

^13^C NMR ([^2^H]-chloroform): δ 152.5 (**C**6), 149.2 (**C**1), 149.1 (**C**5), 143.3 (**C**9), 136.6 (**C**3), 131.5 (**C**10), 129.3 (**C**11), 122.9 (**C**4), 120.2 (**C**12), 118.7 (**C**2), 103.4 (**C**14), 100.0 (**C**7), 64.6 (**C**15), 37.6 (**C**8), 36.7 (**C**13), 15.2 (**C**16).

**4-(4-Bromophenyl)-2,2’-bipyridine (8)**

6.00 g of **7** (16.7 mmol) was dissolved in 200 mL of acetonitrile and 12 g of hydroxylamine hydrochloride (0.173 mol) was added. The mixture was heated under reflux for 10 h. Then the solvent was evaporated, the residue was suspended in dichloromethane (100 mL), and the suspension was washed twice with saturated NaCl solution containing NaOH (1 M). The organic layer was dried over Na_2_SO_4,_ and the solvent was evaporated. The residue was extracted with diethyl ether (4 × 50 mL). The solid material obtained after ether evaporation was crystallized from methanol to obtain **8** as yellowish crystals. Yield: 2.85 g (55%), m.p. 112-113°C.

^1^H NMR ([^2^H]-chloroform): δ 8.73 (dd, 1H, 0.8 & 5.1 Hz; **H**14), 8.71 (ddd, 1H, 1.0, 1.8 & 4.8 Hz; **H**5), 8.64 (dd, 1H, 0.8 & 1.9 Hz; **H**7), 8.45 (dt, 1H, 1.0 & 8.0 Hz; **H**2); 7.84 (ddd, 1H, 1.8, 7.5 & 8.0 Hz; **H**3), 7.63 (m, 4H; **H**10,11), 7.50 (dd, 1H, 1.9 & 5.1 Hz; **H**13), 7.34 (ddd, 1H, 1.0, 4.8 & 7.5 Hz; **H**4).

^13^C NMR ([^2^H]-chloroform): δ 156.7 (**C**6), 155.9 (**C**1), 149.7 (**C**5), 149.1 (**C**14), 148.1 (**C**8), 137.1 (**C**9), 137.0 (**C**3), 132.2 (**C**10), 128.6 (**C**11), 123.9 (**C**4), 123.5 (**C**12), 121.3 (**C**2 or **C**7), 121.2 (**C**2 or **C**7), 118.7 (**C**13).

**4-(4-(4,4,5,5-Tetramethyl-1,3,2-dioxaboran-2-yl)phenyl)-2,2’-bipyridine (9)**

2.00 g of **8** (6.4 mmol), 2.00 g of dried potassium acetate (20.4 mmol), and 2.00 g of bis(pinacolato)diboron (7.9 mmol) was dissolved in dry DMSO (50 mL), and the solution was deoxygenated. Then 150 mg of [1,1′-bis(diphenylphosphino)ferrocene]dichloropalladium(II) (PdCl_2_(dppf); 3.2 mol%) was added and the solution was stirred at 105°C over 24 h. The mixture obtained was extracted with dichloromethane (3 x 200 ml), and the combined organic layers were washed with water. After drying with Na_2_SO_4_, the solvent was evaporated, and the dark solid residue was extracted with hexane (2 x 100 ml). After evaporation, **9** was obtained as a white powder and used without purification. Yield: 1.99 g (87%), m.p. 132-134°C.

^1^H NMR ([^2^H]-chloroform): δ 8.73 (dd, 1H, 0.7 & 5.1 Hz; **H**14), 8.72 (ddd, 1H, 1.0, 1.8 & 4.8 Hz; **H**5), 8.68 (dd, 1H, 0.7 & 1.8 Hz; **H**7), 8.45 (dt, 1H, 1.0 & 7.9 Hz; **H**2), 7.94 (*pseudo*d, 2H; **H**11), 7.84 (td, 1H, 1.8, 7.5 & 7.9 Hz; **H**3), 7,78 (*pseudo*d, 2H; **H**10), 7.57 (dd, 1H, 1.8 & 5.1 Hz; **H**13), 7.34 (ddd, 1H, 1.0, 4.8 & 7.5 Hz; **H**4), 1.38 (s, 12H; **H**16).

^13^C NMR ([^2^H]-chloroform): δ 156.7 (**C**6), 156.1 (**C**1), 149.7 (**C**5), 149.2 (2 x; **C**8,14), 140.8 (**C**9), 137.0 (**C**3), 135.4 (**C**11), ca. 129.5 (broad!; **C**12), 126.4 (**C**10), 123.8 (**C**4), 121.7 (**C**7), 121.3 (**C**2), 119.0 (**C**13), 84.0 (**C**15), 24.9 (**C**16).

| 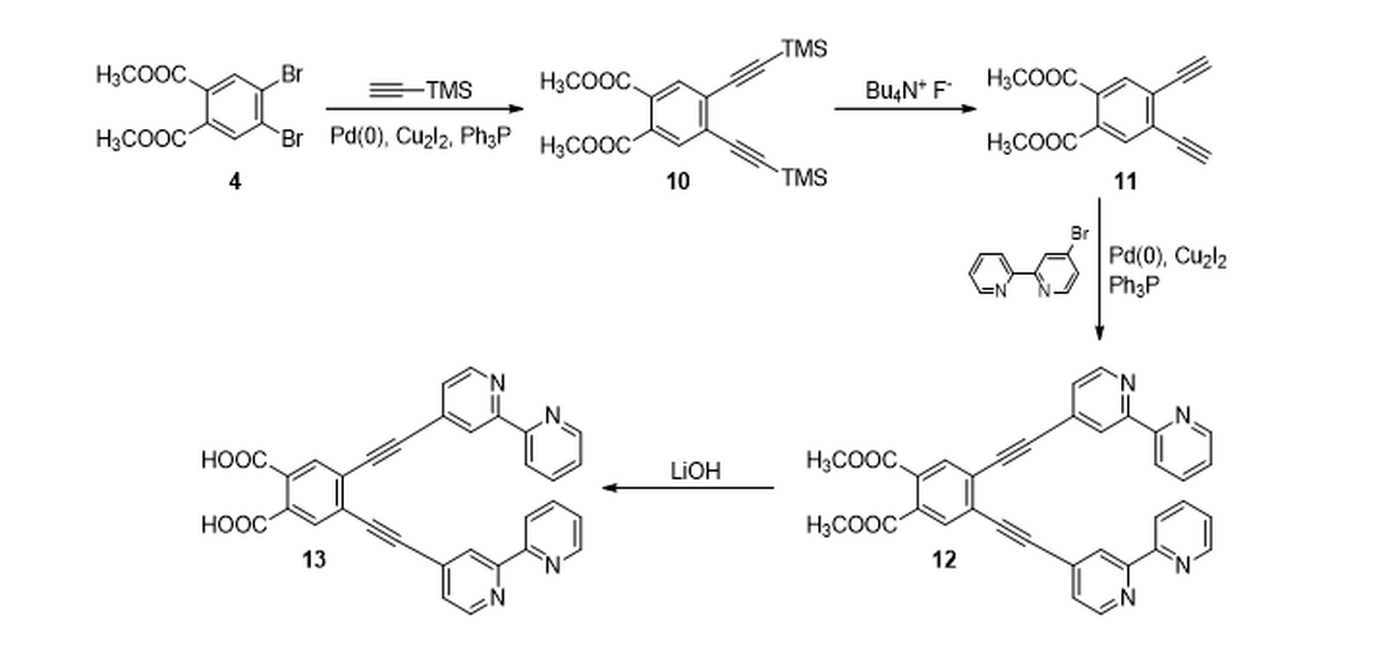 |
| --- |
| Scheme S3. Synthesis of the compounds **10** – **13**. |

**Dimethyl 4,5-bis(trimethylsilylethynyl)phthalate (10)**

Solution of 2.00 g of dimethyl 4,5-dibromophthalate (**4**; 5.7 mmol) and 10 mL of trimethylsilylacetylene (7.1 g; 72.2 mmol) in freshly distilled triethylamine (40 mL) was deoxygenated, and 50 mg Pd_2_(dba)_3_ (1 mol%), 75 mg of triphenylphosphine and 10 mg of copper iodide were added. The obtained mixture was heated over 24 h at 80°C under nitrogen. Then the solvent was evaporated and the residue was dissolved in diethyl ether. The solution was washed with 10% aqueous HCl, dried over Na_2_SO_4_, and evaporated. The product was purified by column chromatography (SiO_2_, dichloromethane) and obtained as white crystals. Yield: 1.91 g (87%), m.p. 178-180°C.

^1^H NMR ([^2^H]-chloroform): δ 7.80 (s, 2H; **H**2), 3.89 (s, 6H; OC**H**_3_), 0.27 (s, 18H; SiC**H**_3_).

^13^C NMR ([^2^H]-chloroform): δ 166.8 (**C**OO), 132.8 (**C**2), 131.0 (**C**1), 128.5 (**C**3), 102.4 (**C**4), 101.5 (**C**5), 52.8 (O**C**H_3_), -0.2 (Si**C**H_3_).

**Dimethyl 4,5-diethynylphthalate (11)**

2.00 g of protected acetylene (**10**; 5.2 mmol) was dissolved in freshly distilled THF (100 mL). Then 0.6 mL of glacial acetic acid (0.63 g; 10.5 mmol) and a solution of tetrabutylammonium fluoride trihydrate (3.5 g; 11.1 mmol) in THF (20 mL) were added. After stirring at room temperature for 1 h solvent was evaporated, and the product was isolated as a white powder by column chromatography (SiO_2_, dichloromethane/diethyl ether 2:1). Yield: 1.22 g (97%), m.p. 97-99°C.

^1^H NMR ([^2^H]-chloroform): δ 7.86 (s, 2H; **H**2), 3.91 (s, 6H; OC**H**_3_), 3.48 (s, 2H; ≡C**H**).

^13^C NMR ([^2^H]-chloroform): δ 166.6 (**C**OO), 133.0 (**C**2), 131.6 (**C**1), 127.9 (**C**3), 84.2 (**C**5), 80.2 (**C**4), 52.9 (O**C**H_3_).

**Dimethyl 4,5-bis(2-(2,2’-bipyridin-4-yl)ethynyl)phthalate (12)**

1.00 g of **11** (4.1 mmol) and 2.10 g of 4-bromo-2,2’-bipyridine (8.9 mmol) were dissolved in deoxygenated 1:1 (v/v) mixture of triethylamine and THF (100 mL). Then 50 mg of Pd_2_(dba)_3_ (2 mol%), 75 mg of triphenylphosphine, and 10 mg of copper iodide were added. The mixture obtained was heated under argon for 24 h at 80°C. After the solvent was evaporated, the product was extracted with chloroform and purified by column chromatography. Yield: 1.96 g (82%) of cream-colored powder, m.p. 248-251°C.

^1^H NMR ([^2^H]-chloroform): δ 8.69 (dd, 2H, 0.8 & 4.9 Hz; **H**10), 8.62 (dd, 2H, 0.8 & 1.6 Hz; **H**7), 8.60 (ddd, 2H, 1.1, 1.8 & 4.7 Hz; **H**15), 8.37 (dt, 2H, 1.1 & 7.9 Hz; **H**13); 7.97 (s, 2H; **H**2), 7.80 (ddd, 2H, 1.7, 7.4 & 7.9 Hz; **H**12), 7.50 (dd, 2H, 1.6 & 4.9 Hz; **H**9), 7.29 (ddd, 2H, 1.1, 4.7 & 7.5 Hz; **H**14), 3.95 (s, 6H; OC**H**_3_).

^13^C NMR ([^2^H]-chloroform): δ 166.6 (**C**OOMe), 156.5 (**C**11), 155.3 (**C**6), 149.4 (**C**15), 149.2 (**C**10), 136.9 (C13), 132.7 (**C**2), 131.9 (**C**1), 131.3 (**C**3), 127.9 (**C**8), 125.3 (**C**7), 124.1 (**C**9), 123.4 (**C**14), 121.1 (**C**12), 94.2 (**C**4), 90.3 (**C**5), 53.0 (O**C**H_3_).

**4,5-Bis(2-(2,2’-bipyridin-4-yl)ethynyl)phthalic acid (13)**

1.50 g of **12** (2.7 mmol) was dissolved in 300 mL of THF and 100 mL of methanol. 0.5 g of lithium hydroxide (21 mmol) in 25 mL of water was added to the solution obtained. The mixture was stirred overnight at room temperature, the solvent was evaporated and the solid residue was suspended in water (50 ml). The solution was neutralized with aqueous 10% HCl, and the precipitated product was filtered off, washed with water and dried. Yield: 1.35 g (95%) of yellowish powder, m.p. 320°C (dec.).

^1^H NMR ([^2^H]-TFA): δ 9.05 (dd, 2H, 1.4 & 5.8 Hz; **H**15), 9.00 (d, 2H, 5.7 Hz; **H**10), 8.80 (td, 2H, 1.4 & 8.0 Hz; **H**13), 8.66 (d, 2H, 8.0 Hz; **H**12), 8.58 (d, 2H, 1.3 Hz; **H**7), 8.29 (s, 2H; **H**2), 8.23 (ddd, 1H, 0.7, 5.8 & 8.0 Hz; **H**14), 8.11 (dd, 1H, 1.3 & 5.6 Hz; **H**9).

^13^C NMR ([^2^H]-TFA): δ 173.4 (**C**OOH), 150.6 (**C**11), 149.4 (**C**6), 146.7 (**C**15), 146.5 (**C**13), 146.0 (**C**10), 141.6 (**C**8), 136.9 (**C**7), 134.3 (**C**9), 132.7 (**C**14), 131.4 (**C**12), 129.8 (**C**2), 129.7 (**C**1), 128.8 (**C**3), 99.2 (**C**4), 93.3 (**C**5).

**Dimethyl 4,5-bis(4-(2,2’-bipirydin-4-yl)phenyl)phthalate (14)**

0.50 g of dibromodiester **4** (1.4 mmol) and 1.20 g of protected boronic acid **9** (3.3 mmol) were dissolved in toluene (80 mL). Then 100 mL of aqueous NaHCO_3_ (5 %) was added, and the mixture was deoxygenated. After the addition of the catalyst, tetrakis(triphenylphosphine)palladium(0) (150 mg; 9 mol%), the mixture was heated over 24 h under reflux. After cooling, the organic layer was separated and the aqueous phase was extracted twice with dichloromethane. Combined organic solutions were dried with Na_2_SO_4_ and evaporated. The product was purified by column chromatography (SiO_2_, dichloromethane/diethyl ether 1:1 (v/v) and obtained as an off-white powder. Yield: 0.67 g (72%), m.p. >300°C (dec.).

^1^H NMR ([^2^H]-chloroform): δ 8.71 (dd, 2H, 0.6 & 5.2 Hz; **H**12), 8.67 (dd, 2H, 1.0, 1.7 & 4.8 Hz; **H**17), 8.66 (dd, 2H, 0.6 & 1.8 Hz; **H**9), 8.44 (dt, 2H, 1.0 & 7.8 Hz; **H**14), 7.87 (s, 2H; **H**2), 7.83 (td, 2H, 1.7 & 7.8 Hz; **H**15), 7.70 (*pseudo*d, 4H; **H**6), 7.54 (dd, 2H, 1.8 & 5.2 Hz; **H**11), 7.32 (m, 6H; **H**5 & **H**16), 3.97 (s, 6H; OC**H**_3_).

| 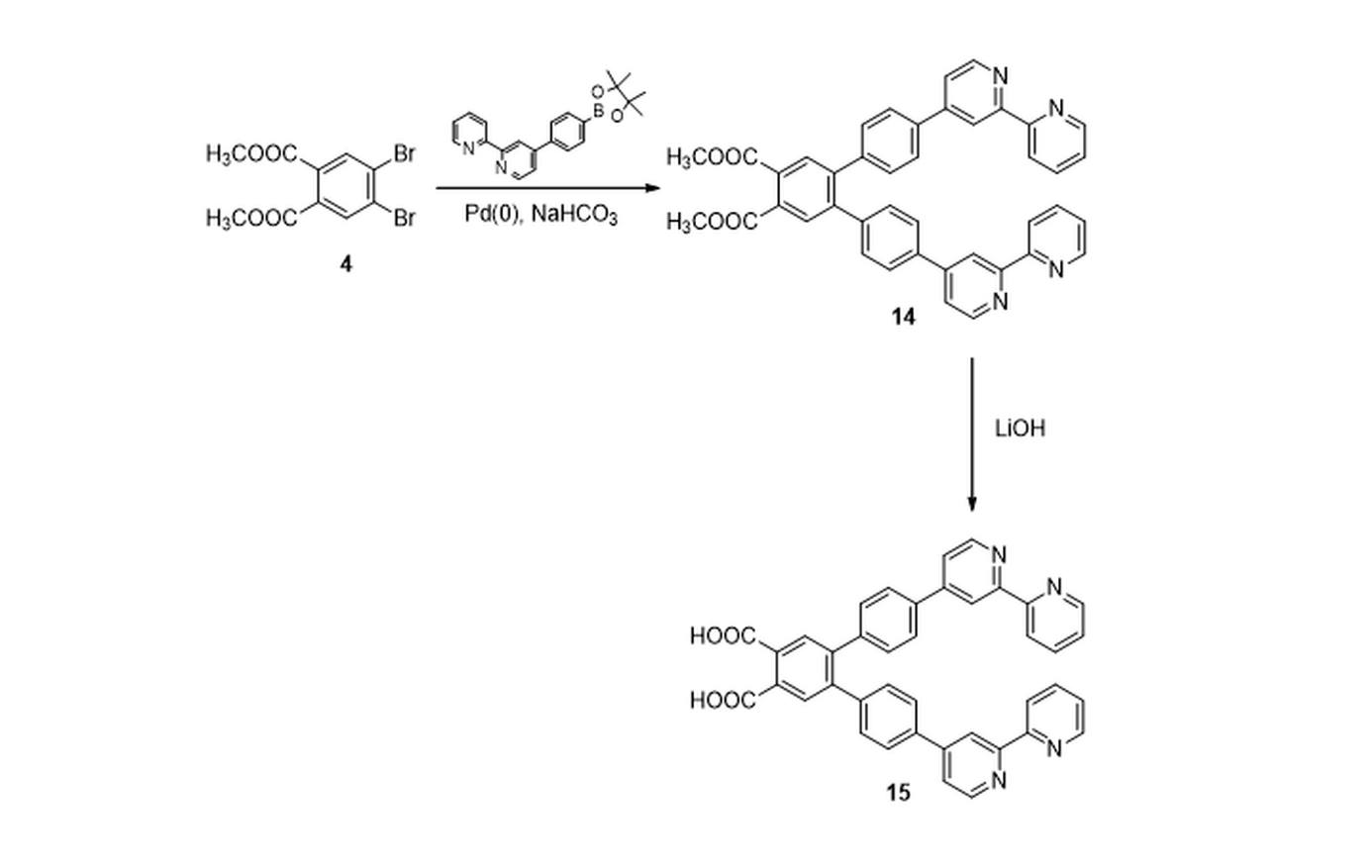 |
| --- |
| Scheme S4. Synthesis route of compounds **14** and **15**. |

**4,5-Bis(4-(2,2’-bipirydin-4-yl)phenyl)phthalic acid (15)**

0.50 g of **14** (0.76 mmol) was dissolved in 300 mL of THF and 100 mL of methanol. 0.5 g of lithium hydroxide (21 mmol) in 25 mL of water was added to the solution obtained. The mixture was stirred overnight at room temperature, the solvent was evaporated and the solid residue was suspended in water (50 ml). The solution was neutralized with aqueous 10% HCl, and the precipitated product was filtered off, washed with water and dried. Yield: 0.44 g (91%) of yellowish powder, m.p. >300°C (dec.).

^1^H NMR ([^2^H]_6_-DMSO): δ 9.89 (s, 2H; COO**H**), 8.72 (dd, 2H, 1.0 & 5.2 Hz; **H**12), 8.70 (d, 2H, 4.9 Hz; **H**17), 8.66 (d, 2H, 1.0 Hz; **H**9), 8.42 (d, 2H, 7.9 Hz; **H**14), 7.96 (t, 2H, 7.9 Hz; **H**15), 7.89 (s, 2H; **H**2), 7.85 (*pseudo*d, 4H; **H**6), 7.78 (d, 2H, 5.2 Hz; **H**11), 7.47 (dd, 2H, 4.9 & 7.9 Hz; **H**16), 7.43 (*pseudo*d, 4H; **H**5).

^13^C NMR ([^2^H]_6_-DMSO): δ 172.6 (**C**OOH), 156.4 (**C**8), 154.0 (**C**13), 149.5 (**C**12 & **C**17), 137.7 (**C**15), 131.9 (**C**1), 133.0 (**C**10), 132.7 (**C**4), 131.9 (**C**6), 131.8 (**C**7), 130.9 (**C**3), 128.1 (**C**5 & **C**2), 126.2 (**C**11), 124.7 (**C**16), 124.1 (**C**14), 120.5 (**C**9).

**B2 dye (16)**

0.25 g of ligand **13** (0.4 mmol) and 0.415 g of *cis*-bis(2,2′-bipyridine)dichlororuthenium(II) dihydrate (0.8 mmol) were dissolved in anhydrous ethanol and heated for 24 h under reflux in an argon atmosphere. The mixture was protected from light. After then, the solid material was filtered off and the solvent was evaporated. The dark brown residue was dissolved in methanol (10 ml), filtered, and a concentrated aqueous solution of ammonium hexafluorophosphate (2 g in 4 mL) was added to the filtrate. The red precipitate formed was filtered off, dissolved in methanol (10 mL), and the precipitation with NH_4_PF_6_ solution was repeated. The product was separated by filtration, washed with water and diethyl ether and dried. B2 dye was obtained as a dark orange amorphous solid, yield: 0,52 g (64%).

Elemental analysis: calcd. for C_80_H_58_N_24_O_4_F_24_P_4_Ru_2_: C, 47.25%; H, 2.88%; N, 8.27%; found: C, 47.09%; H, 2.94%; N, 8.17%.

^1^H NMR ([^2^H]_3_-acetonitrile): δ ~10.8 (bs, 2H; COO**H**), 8.73 (d, 2H, 7.0 Hz; **H**10), 8.63 (s, 2H; **H**2), 8.53 (m, 10H; **H**5’,10’,5”,10”, **H**15), 8.09 (m, 8H; **H**3’,8’,3”,8”), 7.93 (m, 2H; **H**13), 7.75 (m, 12H; **H**2, **H**2’,7’,2”,7” & **H**12), 7.65 (m, 2H; **H**9), 7.50 (m, 2H; **H**7), 7.43 (m, 8H; **H**4’,9’,4”,9”), 7.36 (t, 2H, 7.0 Hz; **H**14).

^13^C NMR ([^2^H]_3_-acetonitrile): δ 170.2 (**C**OOH), 159.4 (**C**11), 158.0 (**C**1’,6’,1”,6”), 157.7 (**C**6), 152.8-152.2 (**C**15, **C**5’,10’,5”,10”), 151.9 (**C**10), 142.8 (**C**13), 140.0-139.0 (C3’,8’,3”,8”), 134.7 (**C**14), 132.0 (**C**9), 129.9 (**C**12), 129.5-129.0 (C4’,9’,4”,9”), 128.7 (**C**1, **C**3), 127.5 (**C**2), 127.0 (**C**8), 126.5 (**C**7), 125.8-125.0 (**C**2’,7’,2”,7”), 94.7 (**C**4), 89.2 (**C**5).

| 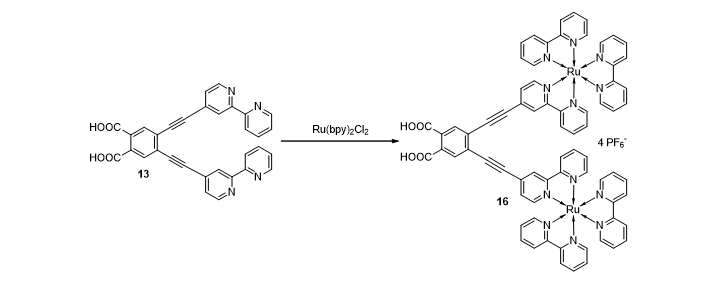 |
| --- |
| Scheme S5. Synthesis of compound **16** (B2 dye). |

**B3 dye (17)**

0.25 g of ligand **15** (0.48 mmol) and 0.498 g of *cis*-bis(2,2′-bipyridine)dichlororuthenium(II) dihydrate (0.96 mmol) were dissolved in anhydrous ethanol and heated for 24 h under reflux in an argon atmosphere. The mixture was protected from light. After then, the solid material was filtered off and the solvent was evaporated. The dark brown residue was dissolved in methanol (15 ml), filtered, and a concentrated aqueous solution of ammonium hexafluorophosphate (2 g in 4 mL) was added to the filtrate. The red precipitate formed was filtered off, dissolved in methanol (15 mL), and the precipitation with NH_4_PF_6_ solution was repeated. The product was separated by filtration, washed with water and diethyl ether and dried. B2 dye was obtained as a dark orange amorphous solid, yield: 0,56 g (73%).

Elemental analysis: calcd. for C_72_H_50_N_12_O_4_F_24_P_4_Ru_2_: C, 44.82%; H, 2.61%; N, 8.71%; found: C, 44.73%; H, 2.68%; N, 8.65%.

^1^H NMR ([^2^H]_3_-acetonitrile): δ ~11.0 (bs, 2H; COO**H**), 9.02 (d, 2H, 5.8 Hz; **H**12), 8.86 (s, 2H; **H**9), 8.65 (d, 2H, 7.1 Hz; **H**17), 8.50 (m, 8H; **H**5’,10’,5”,10”), 8.42 (s, 2H; **H**1), 8.06 (m, 10H; **H**15 & **H**3’,8’,3”,8”), 7.75 (m, 10H; **H**2’,7’,2”,7” & **H**14), 7.69 (*pseudo*d, 4H; **H**6), 7.59 (m, 4H; **H**11 & **H**16), 7.40 (m, 8H; **H**4’, 9’,4”,9”), 7.20 (*pseudo*d, 4H; **H**5).

^13^C NMR ([^2^H]_3_-acetonitrile): δ 169.9 (**C**OOH), 159.1 (**C**8), 158.4 (**C**13), 157.3-157.8 (C1’,6’,1”,6”), 152.7 (**C**12), 151.4 (**C**17), 150.2-150.6 (**C**5’,10’,5”,10”), 141.1 (**C**15), 139.6-138.8 (C3’,8’,3”,8”), 134.1 (**C**7), 133.7 (**C**10), 132.7 (**C**1 & **C**5), 130.9 (**C**4), 129.7 (**C**3), 128.9 (**C**6), 128.6 (**C**2), 127.5 (**C**11), 127.2 (**C**14), 126.5-125.8 (**C**11 & **C**2’,7’,2”,7”), 125.1 (**C**16), 121.0 (**C**9).

| 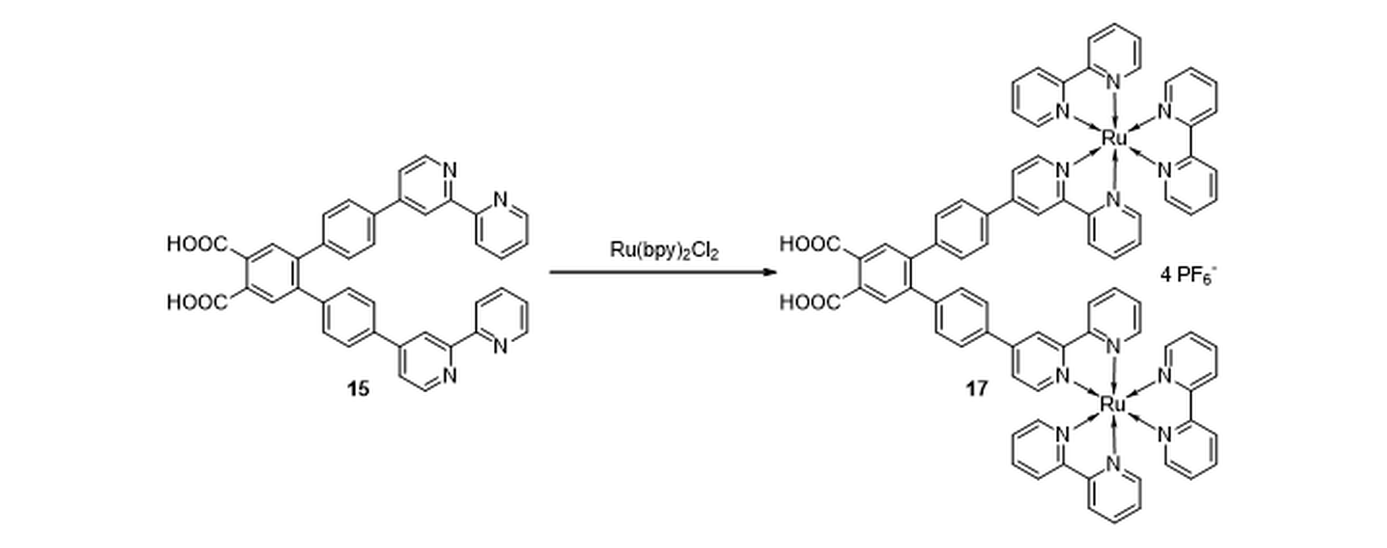 |
| --- |
| Scheme S6. Synthesis of the compound **17** (B3 dye). |


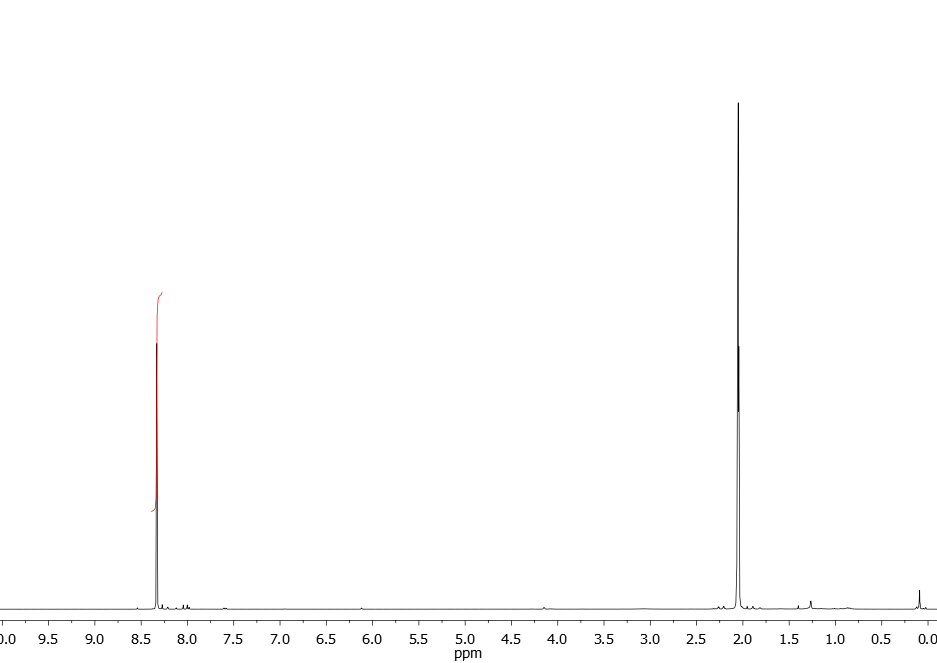


Fig. S5. ^1^H NMR of **2** in [^2^H]_6_-acetone


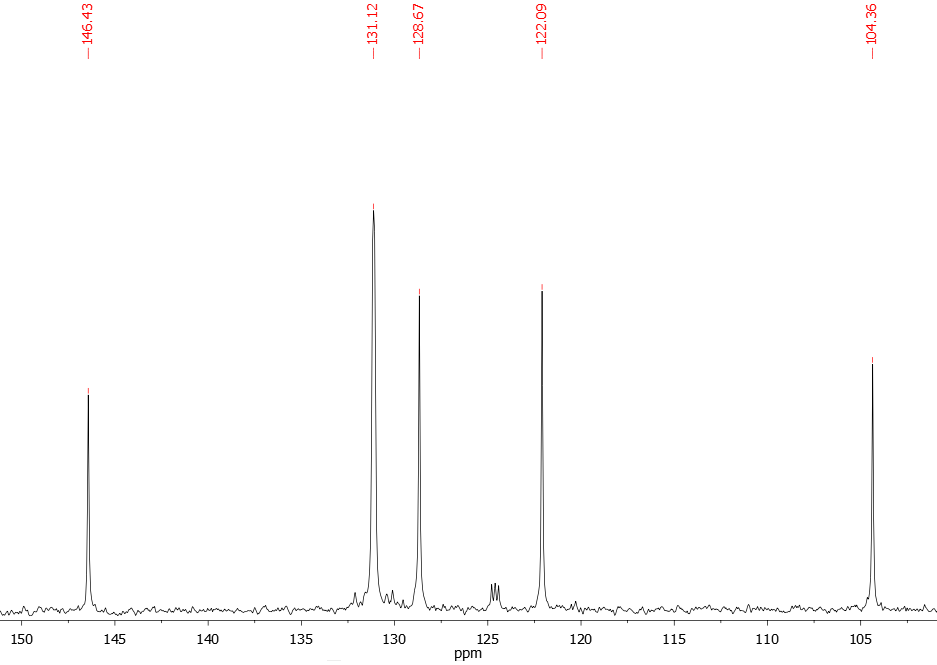


Fig. S6. ^13^C NMR of **2** in [^2^H]_6_-acetone


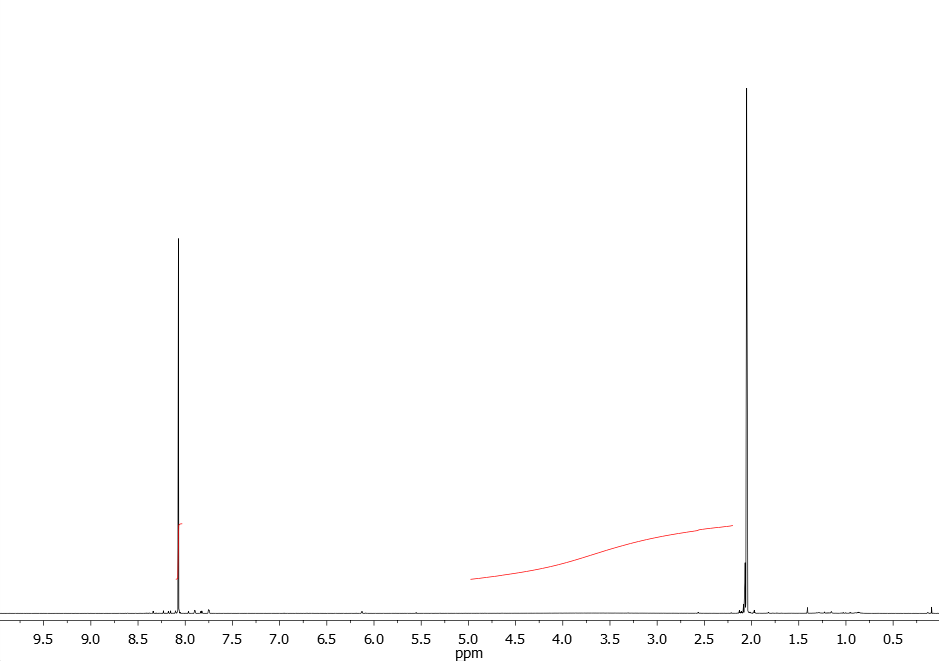


Fig. S7. ^1^H NMR of **3** in [^2^H]_6_-acetone


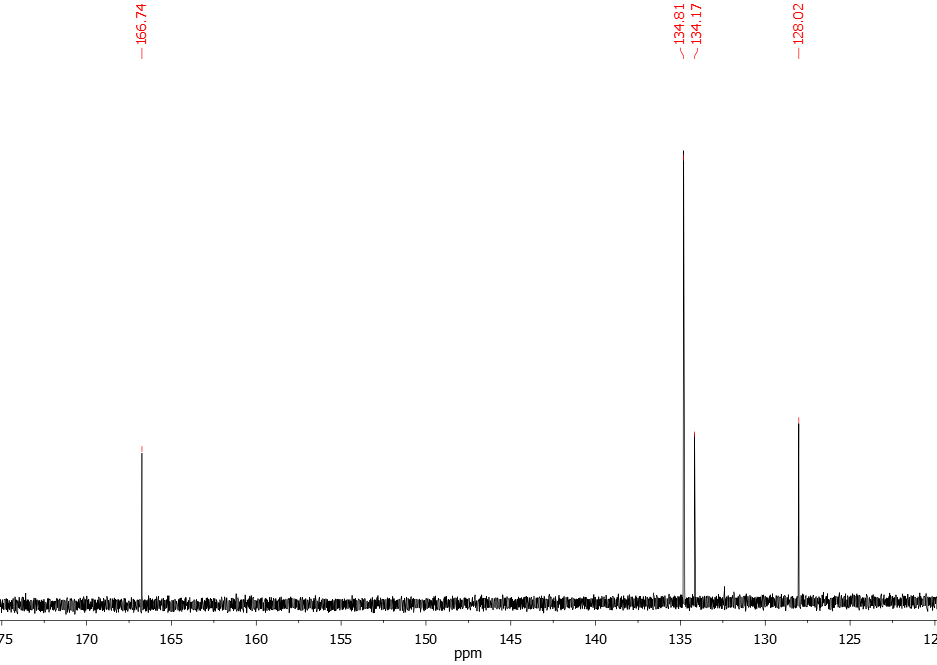


Fig. S8. ^13^C NMR of **3** in [^2^H]_6_-acetone


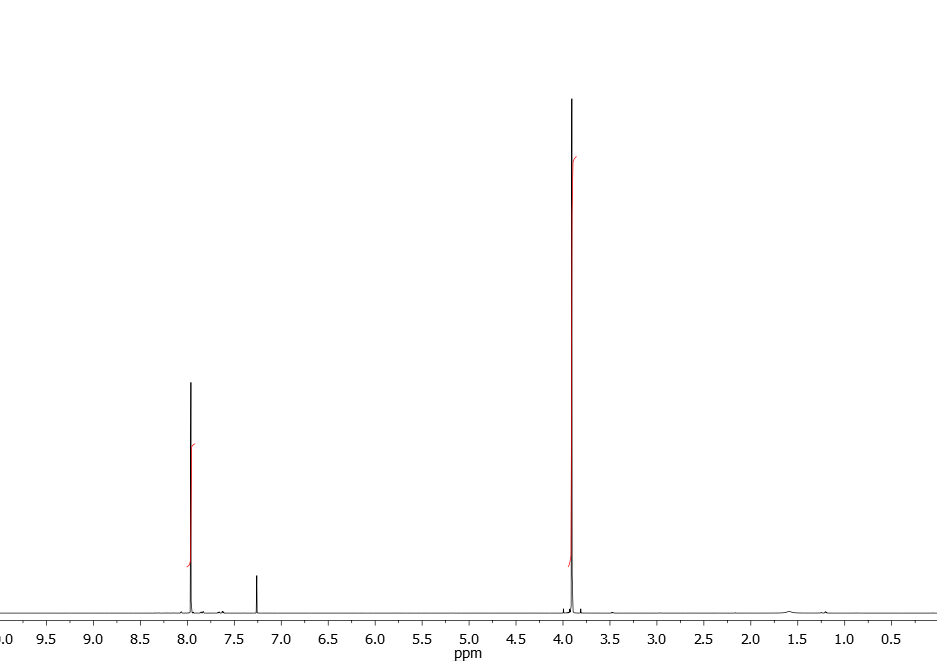


Fig. S9. ^1^H NMR of **4** in [^2^H]-chloroform


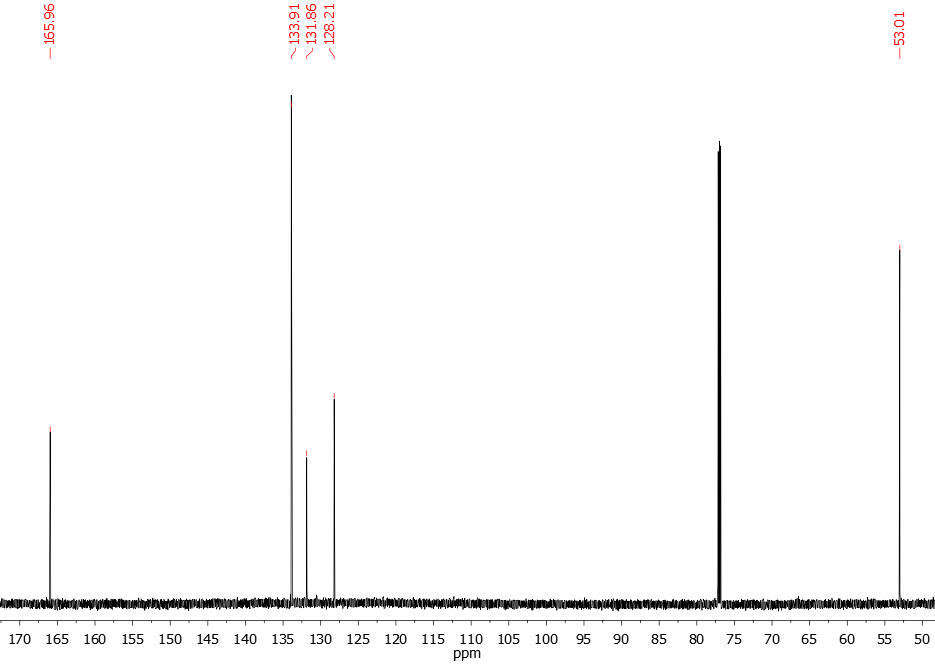


Fig. S10. ^13^C NMR of **4** in [^2^H]-chloroform


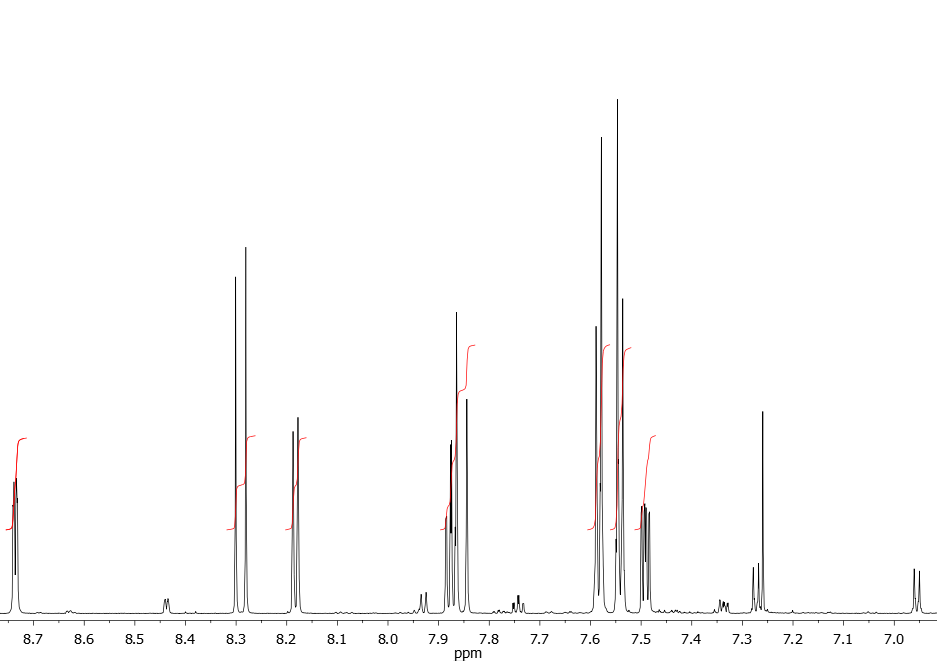


Fig. S11. ^1^H NMR of **6** in [^2^H]-chloroform


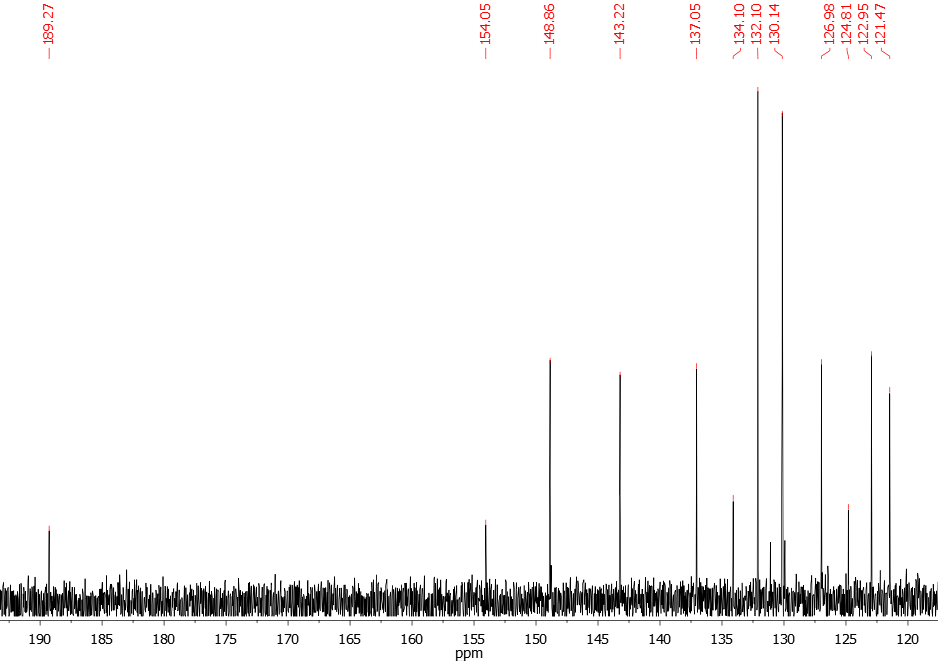


Fig. S12. ^13^C NMR of **6** in [^2^H]-chloroform


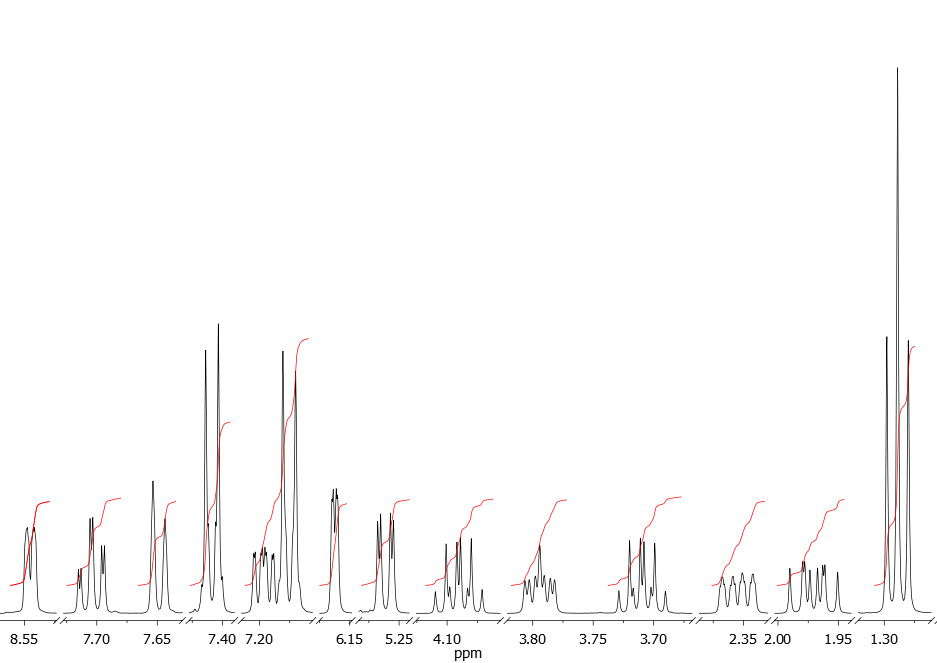


Fig. S13. ^1^H NMR of **7** in [^2^H]-chloroform


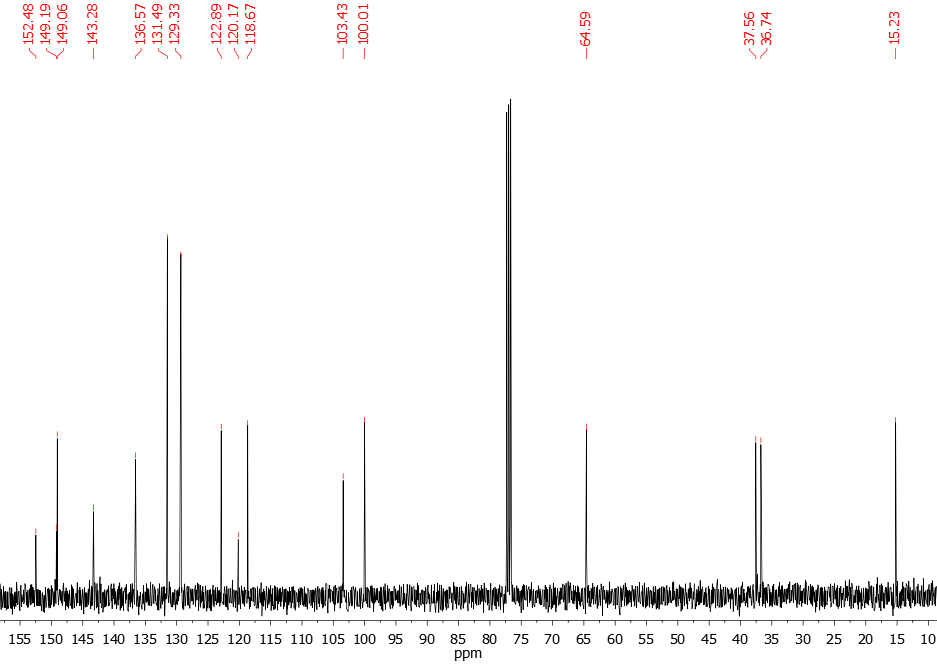


Fig. S14. ^13^C NMR of **7** in [^2^H]-chloroform


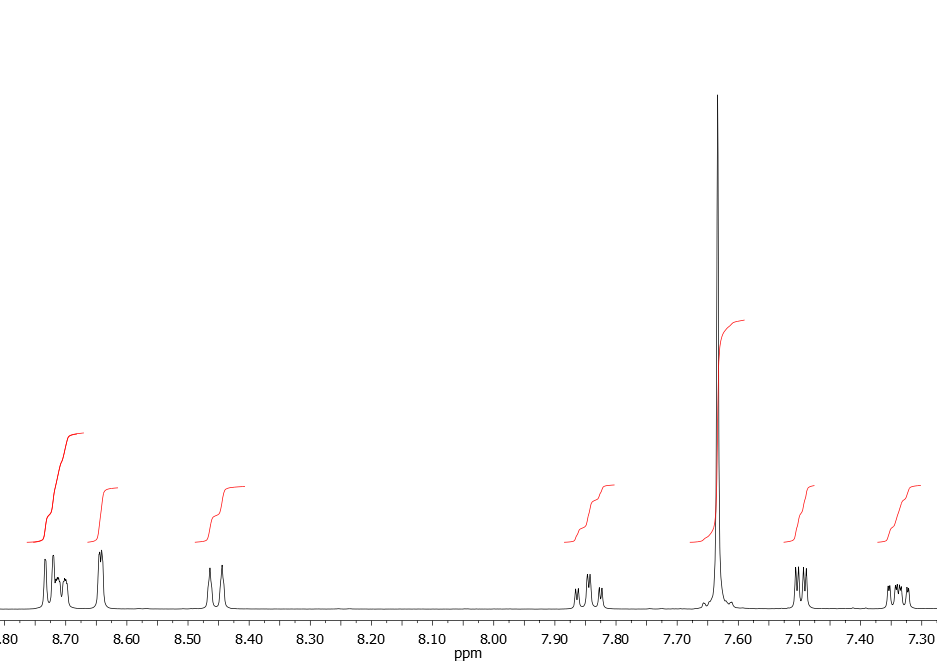


Fig. S15. ^1^H NMR of **8** in [^2^H]-chloroform


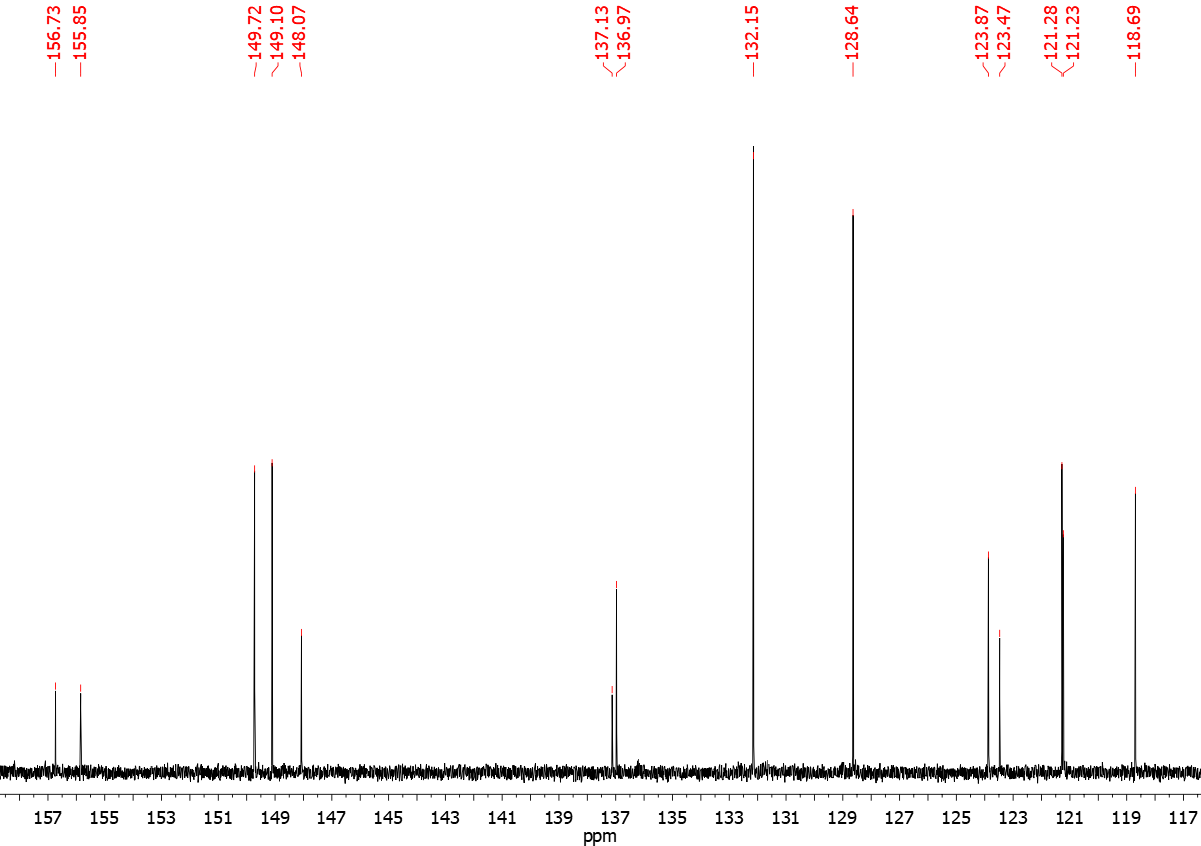


Fig. S16. ^13^C NMR of **8** in [^2^H]-chloroform


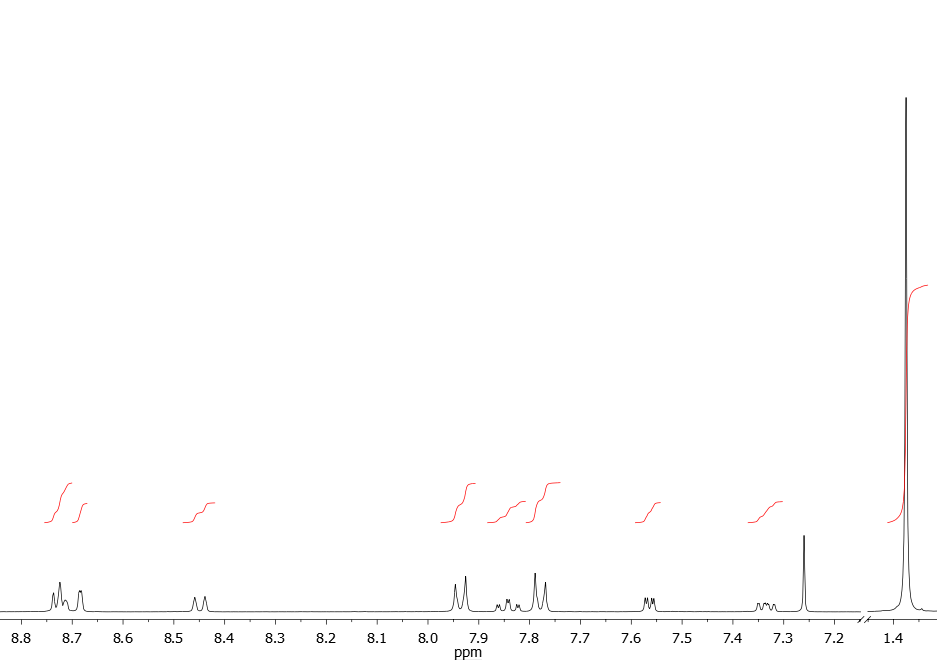


Fig. S17. ^1^H NMR of **9** in [^2^H]-chloroform


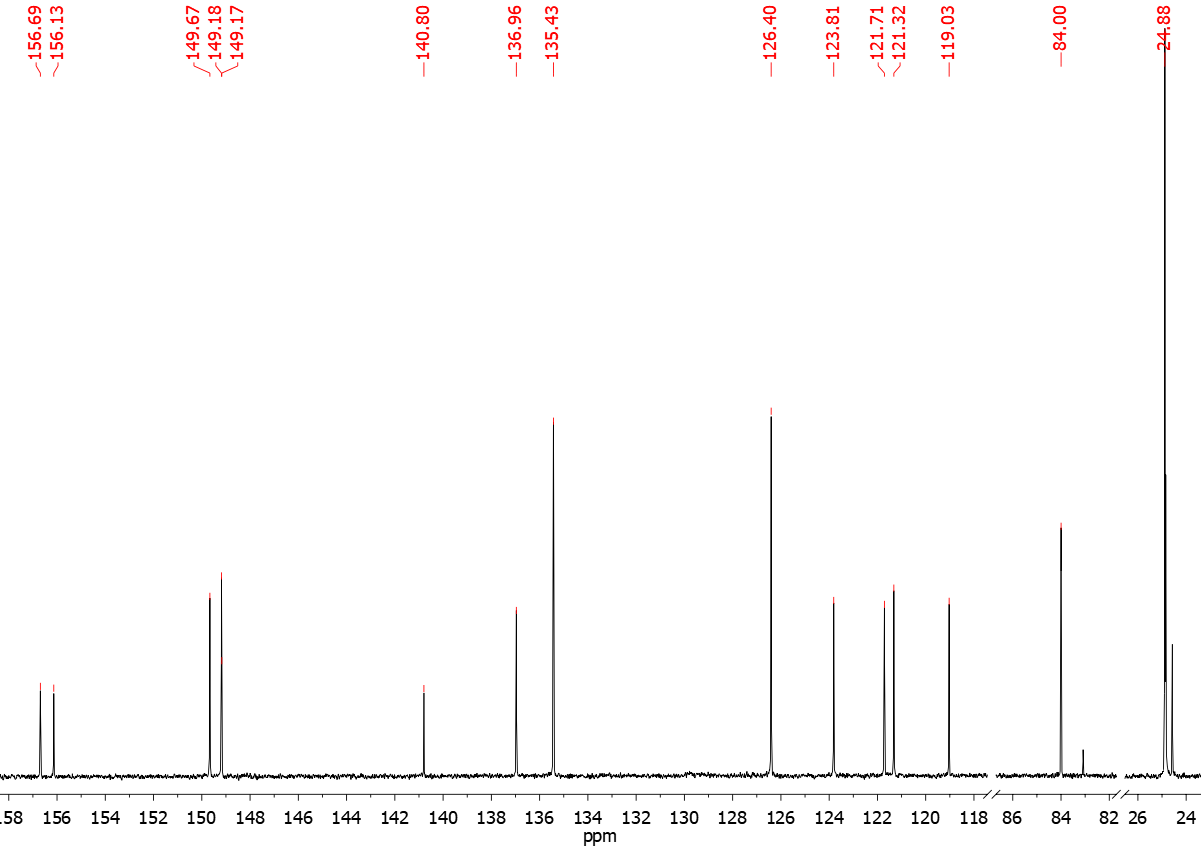


Fig. S18. ^13^C NMR of **9** in [^2^H]-chloroform


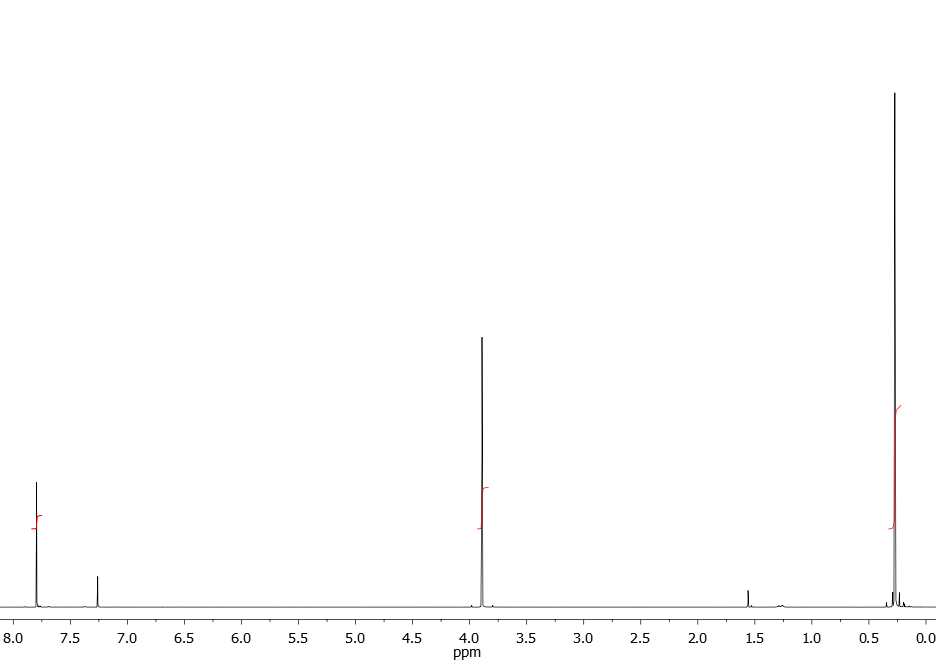


Fig. S19. ^1^H NMR of **10** in [^2^H]-chloroform


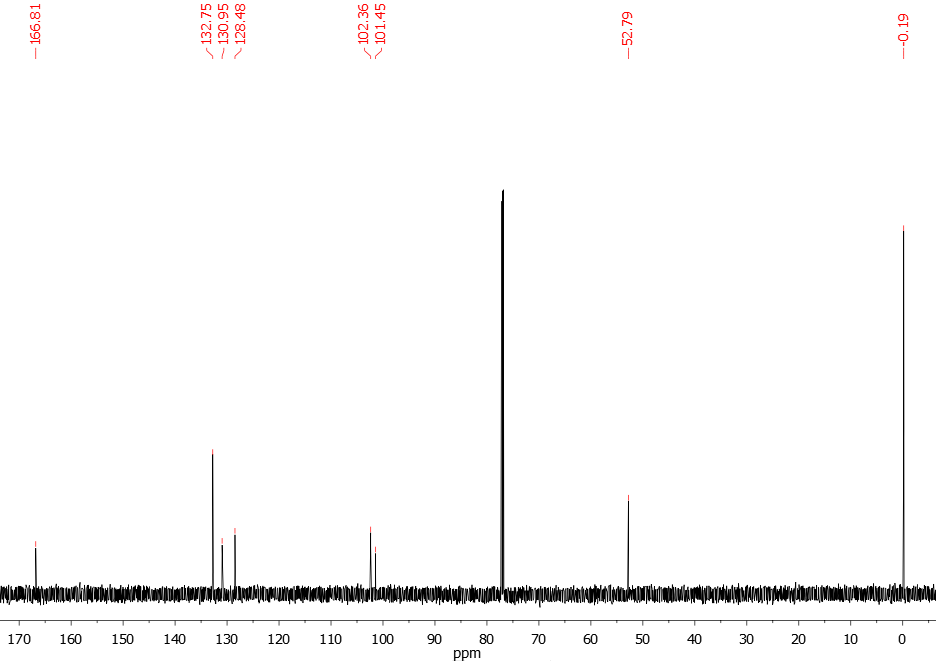


Fig. S20. ^13^C NMR of **10** in [^2^H]-chloroform


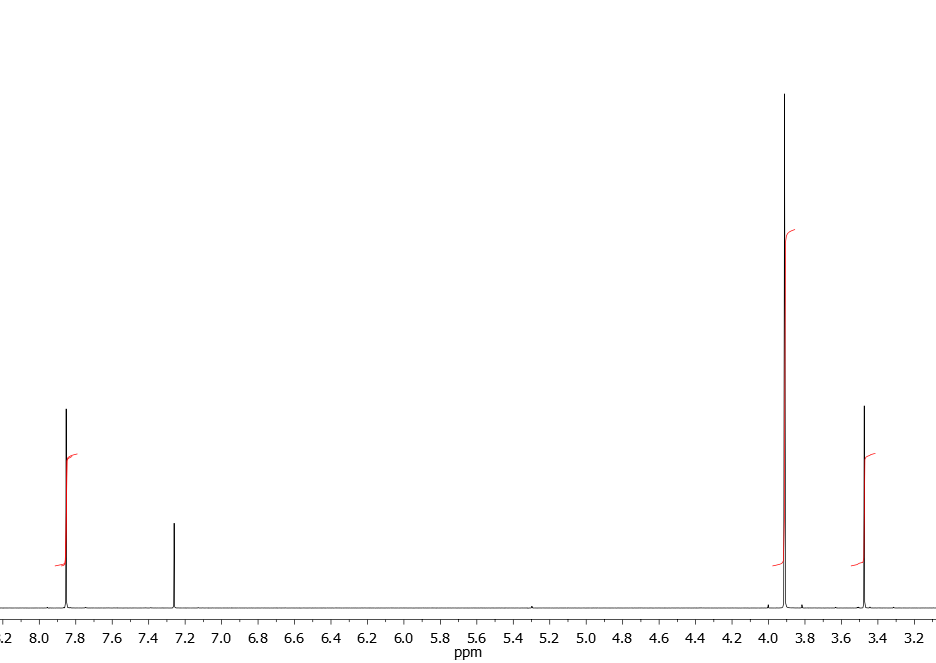


Fig. S21. ^1^H NMR of **11** in [^2^H]-chloroform


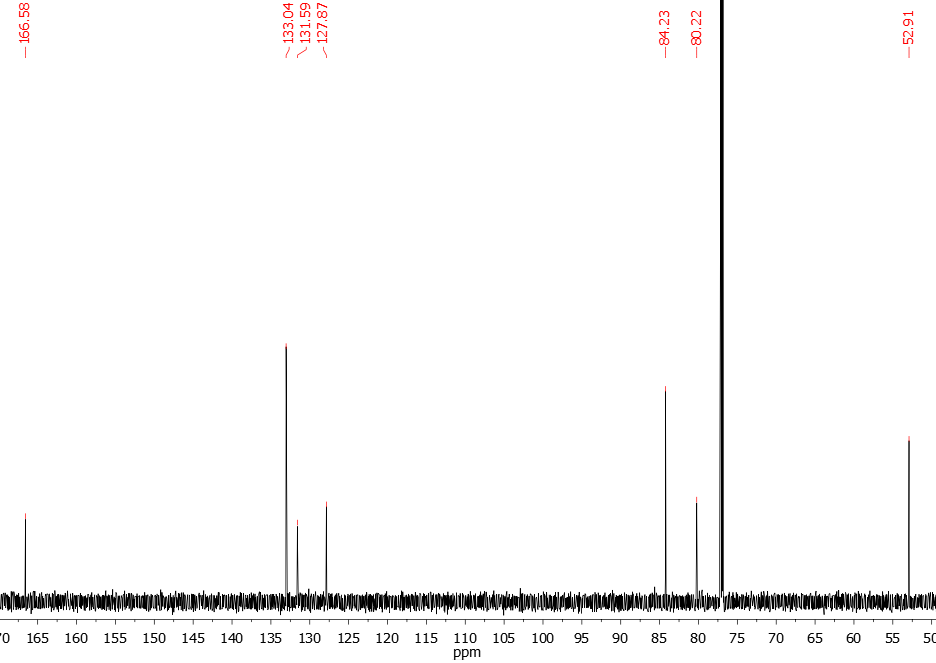


Fig. S22. ^13^C NMR of **11** in [^2^H]-chloroform


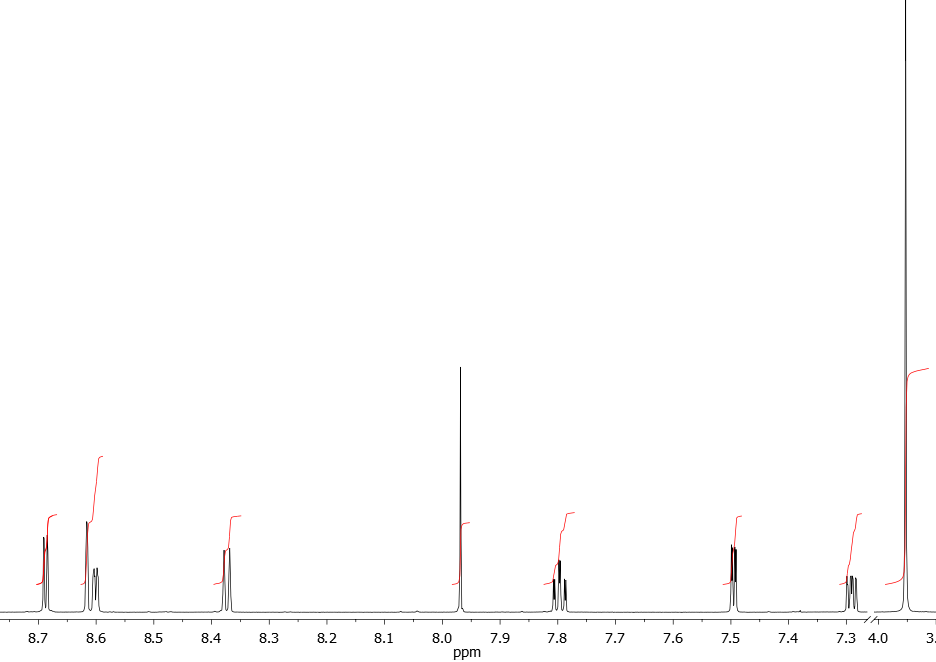


Fig. S23. ^1^H NMR of **12** in [^2^H]-chloroform


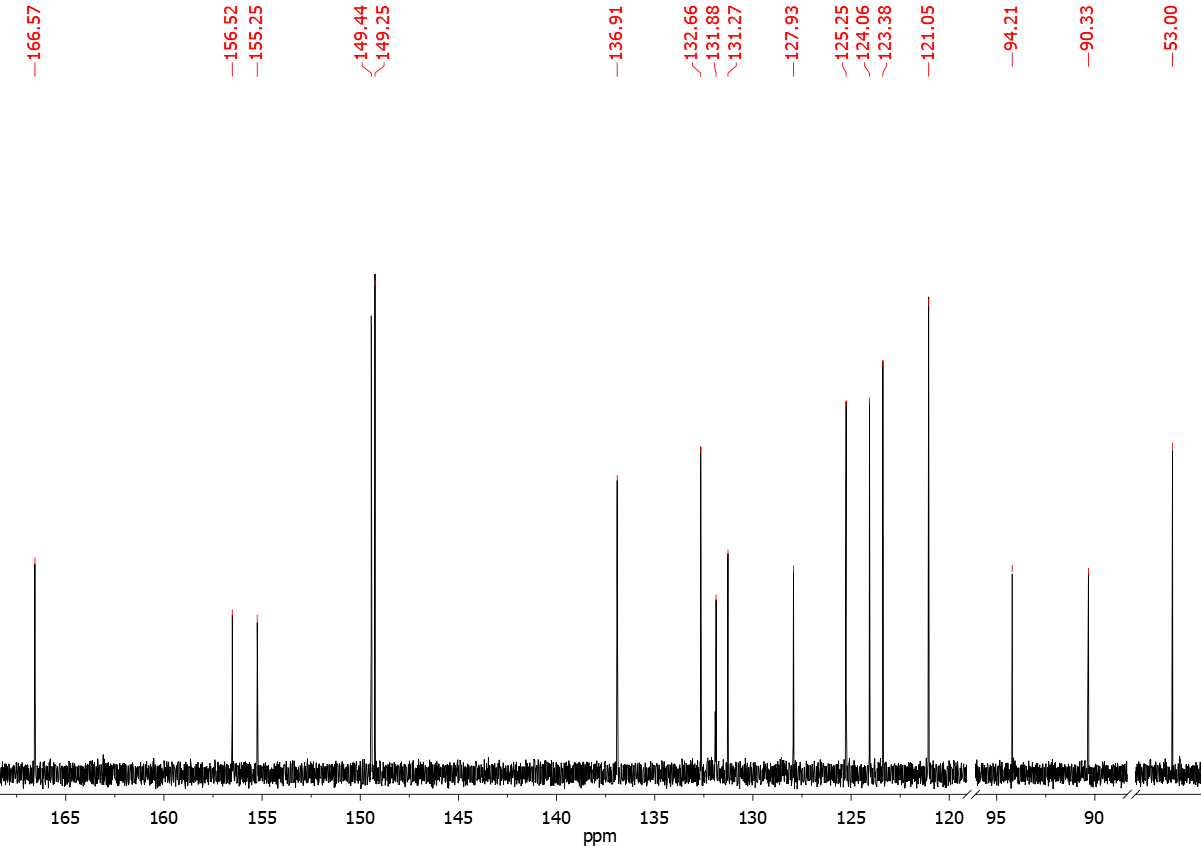


Fig. S24. ^13^C NMR of **12** in [^2^H]-chloroform


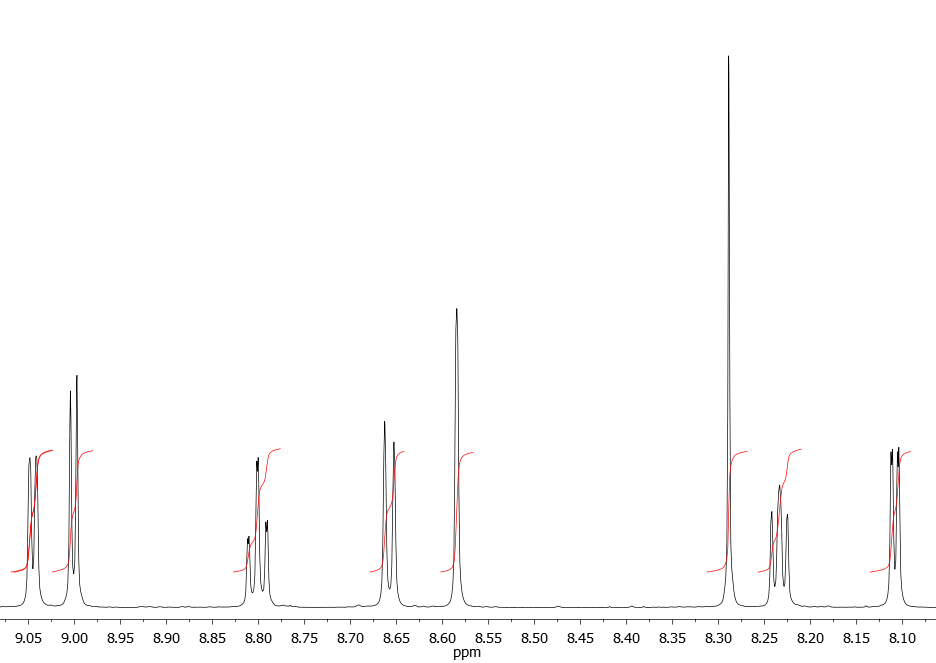


Fig. S25. ^1^H NMR of **13** in [^2^H]-TFA


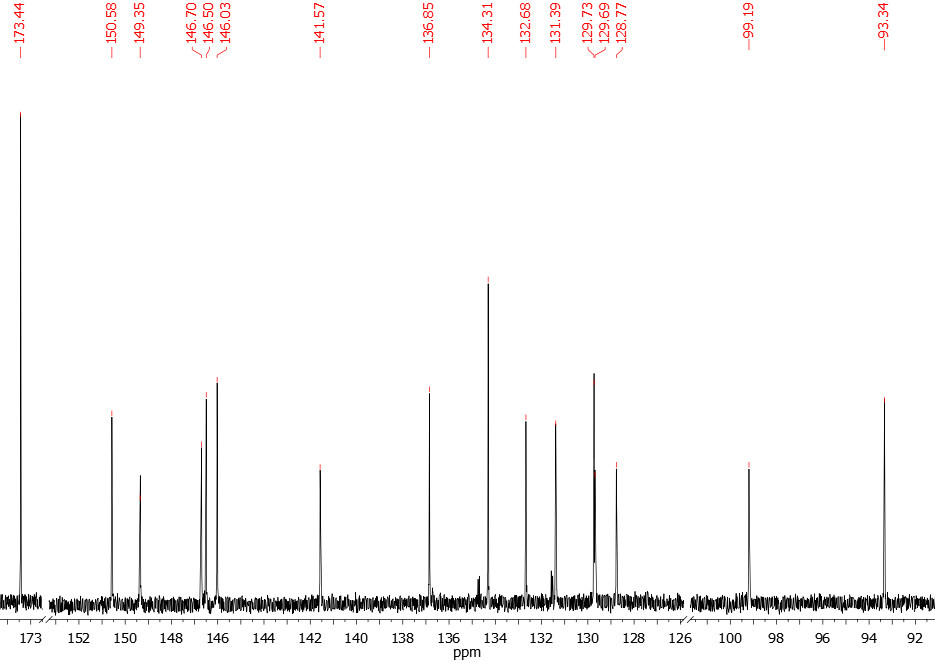


Fig. S26. ^13^C NMR of **13** in [^2^H]-TFA


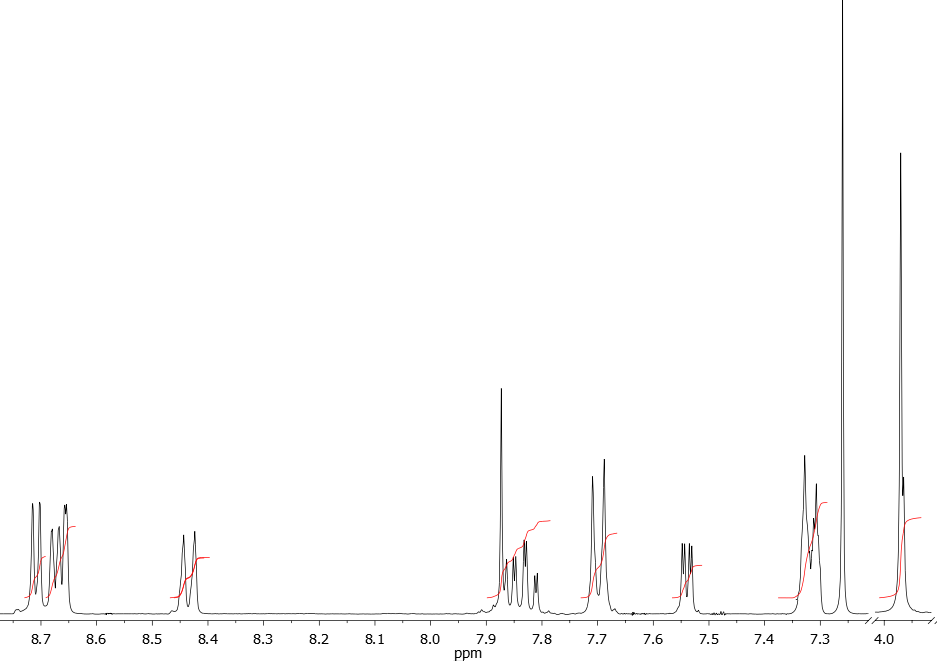


Fig. S27. ^1^H NMR of **14** in [^2^H]-chloroform


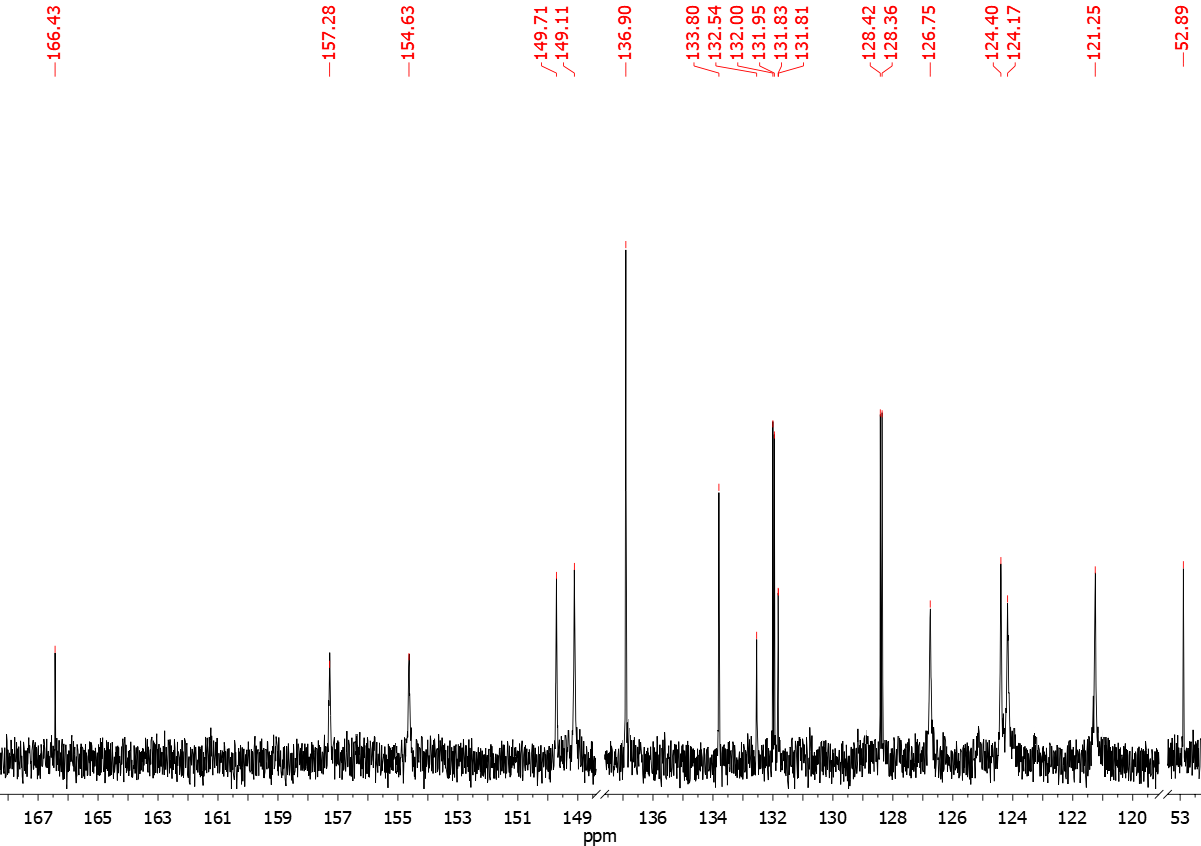


Fig. S28. ^13^C NMR of **14** in [^2^H]-chloroform


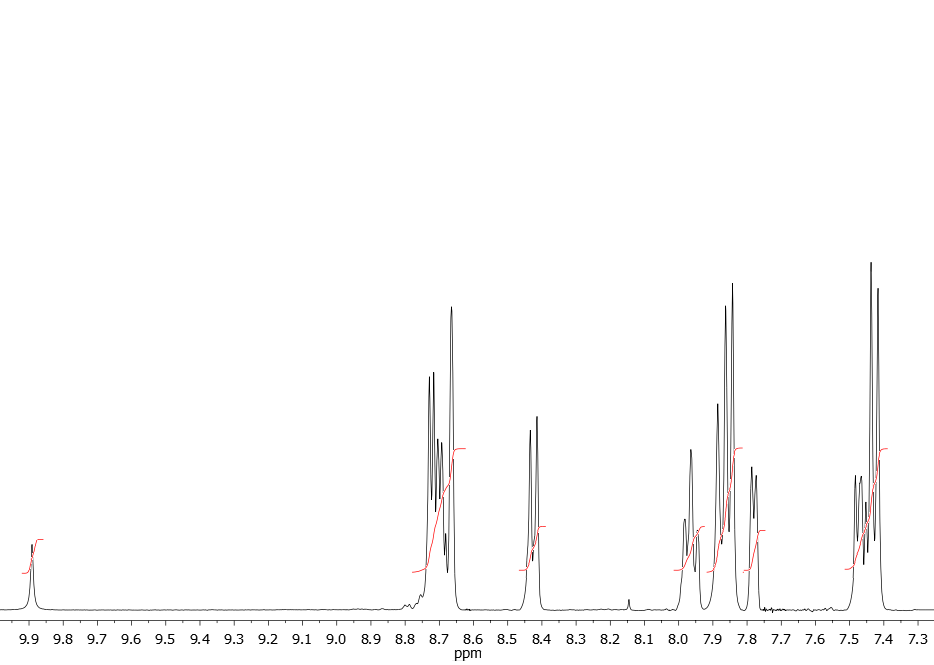


Fig. S29. ^1^H NMR of **15** in [^2^H]_6_-DMSO


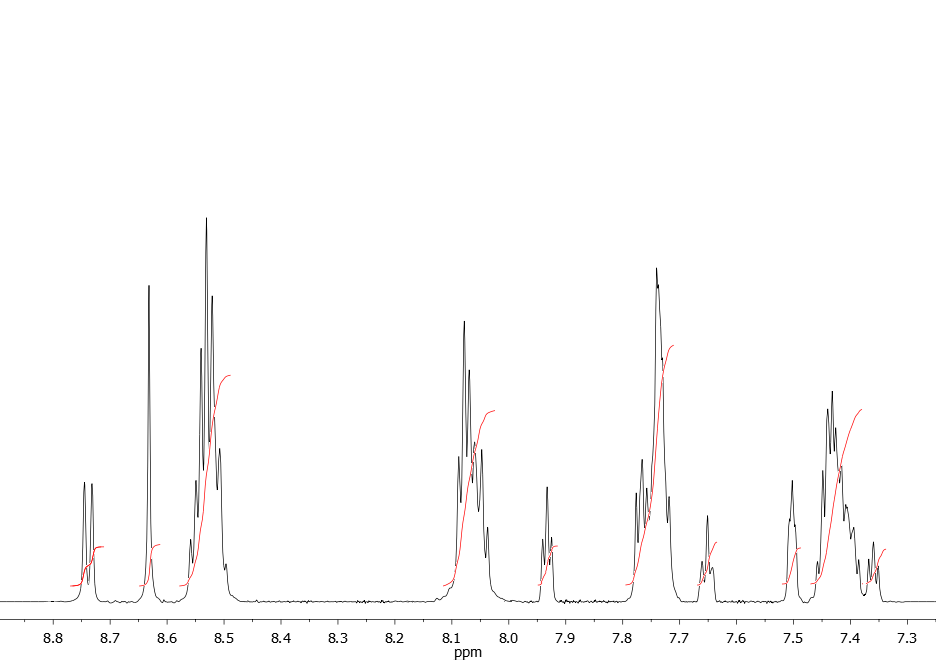


Fig. S30. ^1^H NMR of **16** in [^2^H]_3_-acetonitrile


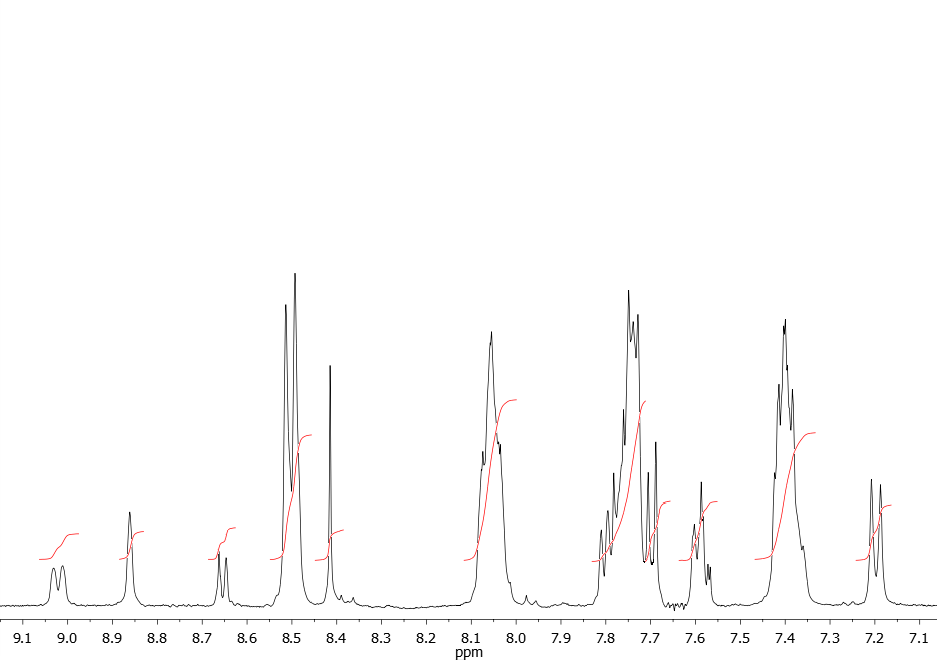


Fig. S31. ^1^H NMR of **17** in [^2^H]_3_-acetonitrile

The octahedral tris(2,2’-bipyridine) ruthenium(II) complex is chiral (*Δ* and *Λ* enantiomers), therefore in monosubstituted Ru(bpy)_3_ unit, all hydrogen and carbon atoms are chemically inequivalent, i.e., differ in chemical shifts. These effects are rather small, therefore, the signals are strongly overlapped and/or broadened and not suitable for detailed analysis and assignments using 2D NMR techniques (for example, see the doubled C^p^ signal for Ru(bpy)_2_(bpyCOOH))^3^. The situation is more complicated in studied two-center complexes since the molecules formed are diastereomeric. Dyes B2 and B3 are mixtures of two enantiomers. i.e. *Δ*,*Δ* and *Λ*,*Λ*-isomers and its *meso*-form (diastereoisomer): *Δ*,*Λ*. Chemical shifts for enantiomers are the same in non-chiral solutions; however, one could expect small differences in *δ* values for diastereoisomers. These isomers are inseparable by chromatographic methods. Although free 2,2-bipyridine molecules are symmetrical, the maximum number of signals for bpy units in B2 and B3 is eight for each type of carbon or hydrogen atom (e.g., C^2^), because the formation of two diastereoisomers and chemical inequivalence of all atoms. This results in somewhat “inelegant” spectra – some signals, the most affected by the effects mentioned above, are broadened, asymmetric, or even divided into several tightly located lines.

**References**

1. Zalas, M. *et al.* Synthesis of a novel dinuclear ruthenium polypyridine dye for dye-sensitized solar cells application. *Polyhedron* **67**, (2014).

2. Zalas, M. *et al.* Erratum to “Synthesis of a novel dinuclear ruthenium polypyridine dye for dye-sensitized solar cells application” [Polyhedron 67 (2014) 381–387](S0277538713006840)(10.1016/j.poly.2013.09.023). *Polyhedron* (2019) doi:10.1016/j.poly.2019.05.041.

3. Zhou, M., Robertson, G. P. & Roovers, J. Comparative study of ruthenium(II) tris(bipyridine) derivatives for electrochemiluminescence application. *Inorg Chem* **44**, 8317–8325 (2005).

4. Welter, S. *et al.* Rodlike bimetallic ruthenium and osmium complexes bridged by phenylene spacers. Synthesis, electrochemistry, and photophysics. *Inorg Chem* **44**, 4706–4718 (2005).

5. Cordaro, J. G., McCusker, J. K. & Bergman, R. G. Synthesis of monosubstituted 2,2′-bipyridines. *Chemical Communications* **2**, 1496–1497 (2002).
